# Rapid phenotypic evolution in multidrug-resistant *Klebsiella pneumoniae* hospital outbreak strains

**DOI:** 10.1099/mgen.0.000263

**Published:** 2019-04-02

**Authors:** Lucy van Dorp, Qi Wang, Liam P. Shaw, Mislav Acman, Ola B. Brynildsrud, Vegard Eldholm, Ruobing Wang, Hua Gao, Yuyao Yin, Hongbin Chen, Chuling Ding, Rhys A. Farrer, Xavier Didelot, Francois Balloux, Hui Wang

**Affiliations:** ^1^​ UCL Genetics Institute, University College London, Gower Street, London WC1E 6BT, UK; ^2^​ Department of Clinical Laboratory, Peking University People's Hospital, Beijing, 100044, PR China; ^3^​ Nuffield Department of Medicine, John Radcliffe Hospital, Oxford OX3 7BN, UK; ^4^​ Infectious Diseases and Environmental Health, Norwegian Institute of Public Health, Lovisenberggata 8, 0456, Oslo, Norway; ^5^​ Medical Research Council Centre for Medical Mycology at the University of Aberdeen, Aberdeen Fungal Group, Institute of Medical Sciences, Foresterhill, Aberdeen AB25 2ZD, UK; ^6^​ School of Life Sciences and the Department of Statistics, University of Warwick, Coventry CV4 7AL, UK

**Keywords:** antimicrobial resistance, carbapenem-resistant *Klebsiella pneumoniae* (CRKP), horizontal gene transfer, nosocomial pathogens, plasmids, transmission chains

## Abstract

Carbapenem-resistant *Klebsiella pneumoniae* (CRKP) increasingly cause high-mortality outbreaks in hospital settings globally. Following a patient fatality at a hospital in Beijing due to a *bla*_KPC-2_-positive CRKP infection, close monitoring was put in place over the course of 14 months to characterize all *bla*_KPC-2_-positive CRKP in circulation in the hospital. Whole genome sequences were generated for 100 isolates from *bla*_KPC-2_-positive isolates from infected patients, carriers and the hospital environment. Phylogenetic analyses identified a closely related cluster of 82 sequence type 11 (ST11) isolates circulating in the hospital for at least a year prior to admission of the index patient. The majority of inferred transmissions for these isolates involved patients in intensive care units. Whilst the 82 ST11 isolates collected during the surveillance effort all had closely related chromosomes, we observed extensive diversity in their antimicrobial resistance (AMR) phenotypes. We were able to reconstruct the major genomic changes underpinning this variation in AMR profiles, including multiple gains and losses of entire plasmids and recombination events between plasmids, including transposition of *bla*_KPC-2_. We also identified specific cases where variation in plasmid copy number correlated with the level of phenotypic resistance to drugs, suggesting that the number of resistance elements carried by a strain may play a role in determining the level of AMR. Our findings highlight the epidemiological value of whole genome sequencing for investigating multi-drug-resistant hospital infections and illustrate that standard typing schemes cannot capture the extraordinarily fast genome evolution of CRKP isolates.

## Data Summary

Raw Illumina short-read and PacBio long-read sequences have been deposited on NCBI with BioProject ID PRJNA508760. Full accompanying metadata and phenotypic resistance characterization are available in Tables S1 and S2 (available in the online version of this article). All programs used in bioinformatics analyses are publicly available.

**Impact Statement**

Multi-drug-resistant bacteria cause high-mortality disease outbreaks in hospitals worldwide. We set up a monitoring program in a hospital in Beijing following the death of a patient due to an infection with a *Klebsiella pneumoniae* strain resistant to multiple drugs, including to carbapenems, the last-resort antibiotics. We isolated 100 carbapenem-resistant *Klebsiella pneumoniae* (CRKP) swabbed from infected patients, patient carriers and the hospital environment over 14 months. We generated complete genomes for all strains and reconstructed their evolution and transmission within the hospital. The majority of the CRKP isolates belonged to a single outbreak which started spreading at least one year before the monitoring program began. While these bacteria had accumulated little genetic variation on their chromosomes, they carried extraordinary variation in drug resistance and virulence genes. We show that this diversity was generated during the course of the outbreak through frequent gains and losses of plasmids and exchange of genetic material between plasmids. We also identified cases where the number of copies of a plasmid predicted the level of drug resistance. Our results showcase the value of genome sequencing for investigating multi-drug-resistant hospital outbreaks but highlight that standard bioinformatics analyses are unable to capture the extraordinary rapid genomic evolution of CRKPs.

## Introduction

The increasing incidence of antimicrobial resistance (AMR) in nosocomial bacteria poses a growing threat to patient populations in hospitals worldwide. The genetic basis underlying AMR phenotypes varies depending on the therapeutic drug class, resistance mechanism and organism. Some forms of AMR are conferred by mutations in the shared core genome, and are generally located on the bacterial chromosome. Conversely, other types of resistance are determined by variation in the composition of the accessory genome, through the presence/absence of mobile resistance genetic elements acquired by horizontal gene transfer (HGT).

Whole genome sequencing (WGS) allows the characterization of the full genomic repertoire of nosocomial bacteria, accounting for both the core and the accessory components. Beyond its uses for strain identification and outbreak tracking, WGS can be used to predict phenotypic traits encoded by non-core genes. As a result, WGS-based resistotyping can be as accurate as, if not superior to, traditional approaches [1–3]. Phenotype prediction from WGS is particularly relevant for traits such as AMR and virulence, which are often encoded on conjugative plasmids that readily pass between strains in circulation in hospital wards. As a consequence, bacteria with open genomes and/or high plasmid carriage comprise some of the most clinically challenging species worldwide [4].

Amongst the most concerning examples of multi-drug-resistant (MDR) hospital pathogens are carbapenem-resistant *Klebsiella pneumoniae* (CRKP) [5]. Carbapenems, such as imipenem and meropenem, are a class of antibiotics widely used in the treatment of severe infections caused by *Enterobacteriaceae* producing extended-spectrum β-lactamases (ESBLs). With few therapeutic options remaining, CRKP infections are often associated with high levels of morbidity and mortality [6, 7].

Carbapenem resistance in *K. pneumoniae* is most commonly mediated by the *bla*_KPC-2_ gene, which was first identified in 1996 in the USA and is now endemic globally, with the first record in China dating back to 2004 [8–10]. *bla*_KPC-2_ is located on a Tn3-family transposon that can insert in multiple places in the genome. *bla*_KPC-2_ can therefore be transmitted clonally via the chromosome [11, 12], but is more commonly found on plasmids [13–17], including those harbouring other significant AMR- [18–20] and virulence-associated genes [21, 22]. The ability of the *bla*_KPC-2_ transposon to insert and relocate between plasmids, across multiple enterobacterial species [16, 23–25], suggests that the selective pressures in hospital environments create an ideal set of conditions for highly transmissible forms of AMR [13, 16, 26–31]. During outbreaks, CRKP can readily spread between clinically diagnosed infected patients, but also has the potential to be acquired and spread via colonized medical equipment and asymptomatic patient or staff carriers. This is particularly concerning given evidence for prolonged CRKP colonization in hospital settings [32].

Whilst several CRKP outbreaks have been reported in the literature [23, 33–36], the complex drivers of transmission and the role of plasmid and transposon dynamics in shaping the genetic and phenotypic diversity of strains remains challenging to resolve. This prompted us to set up a surveillance initiative for CRKP isolates over a 14-month period at Peking University People’s Hospital (PKUPH), Beijing. Using the rich information provided by WGS we investigated the full genomic diversity of *bla*_KPC-2_ isolates in circulation, characterized the genetic context of resistance, and reconstructed transmission pathways within the hospital by integrating genomic and epidemiological data.

## Methods

### Data collection and description of isolates

A hospital-wide initiative was set up in February 2016 at PKUPH to manage and characterize a CRKP outbreak. All patients newly admitted to wards with known prevalence of CRKP had rectal, throat or axillary swabs taken within 24 h of their admission. Swabbing of the environment was also performed on wards where CRKP had been detected. This effort spanned three hospital campuses and 19 wards, including two intensive care units (Figs S1 and S2). All swabs, including those taken from the infection sites of diagnosed CRKP-infected patients, were plated and screened using China Blue Agar containing meropenem at 1 µg ml^−1^. The presence of *bla*_KPC_ was confirmed through PCR. PCR products were visualized by agarose gel electrophoresis, purified with a QIAquick PCR Purification Kit (Qiagen) and sequenced by Sanger sequencing on an ABI PRISM 3730XL system (Applied Biosystems).

All PCR-positive *bla*_KPC-2_ strains were retained for WGS analysis. Additionally, we also included strains that were *bla*_KPC-2_-negative as long as they had been sampled from a patient who was also infected with a *bla*_KPC-2_-positive strain during the surveillance period (isolates CX48 and CX106). This resulted in a final dataset of 100 isolates collected over a 14-month period from: (i) the infection sites of clinically diagnosed patients who were sampled at a single time point or sequentially through their hospital admission (*n*=56), (ii) in-patient carriers identified by standard throat, nose, axillary and rectal swab screens within 24 h of their admission (*n*=18), (iii) patients who were positive for CRKP at both the site of clinical infection and from surveillance screening swabs (*n*=15), and (iv) from patient-associated ward environments, including medical equipment and bed rails (*n*=11) (Table S1 and Fig. S3).

Total DNA was extracted using the TIANamp Bacteria DNA Kit DP302 (Tiangen Biotech) and stored at −80 °C. All isolates were sequenced using the Illumina Hiseq 2500 system. Eight isolates were also later sequenced by the PacBio RS II system (Pacific Biosciences) with a 10-kb size library and P6/C4 chemistry. All isolates were also identified by MALDI-TOF and their phenotypic resistance to several antibacterial agents was determined by minimal inhibitory concentrations (MICs) through agar or broth dilution methods (Table S2). Following the 14-month surveillance period, the CRKP outbreak was brought under control. This was aided by hospital-wide interventions, including enhanced environmental cleaning of CRKP-infected wards and the establishment of a new air disinfection protocol.

### Reference-based whole genome alignment and variant calling

We ran the genomic distance estimation tool Mash [37] on trimmed and quality-filtered Illumina raw sequencing reads to identify the best matching chromosomal reference against an archive of complete *K. pneumoniae* genomes from NCBI RefSeq. The best-matching chromosomal reference was *K. pneumoniae* strain SWU01 (NZ_CP018454.1). Reads were mapped to this reference using bwa-mem v0.7.12 [38] and SNPs were called independently for each sample using FreeBayes v1.0.2 [39], with a minimum alternative fraction of 0.95 and otherwise default parameters. Additional SNP filtering was then performed in bcftools to extract only those SNPs with a Phred-scaled quality score of at least 50. Regions of recombination in the resulting alignment were identified and excluded using ClonalFrameML v1.11 [40]. This left a 5 057 386 bp alignment comprising 458 SNPs across the 82 closely related sequence type 11 (ST11) isolates. A maximum-likelihood phylogenetic tree was inferred from this recombination-free chromosomal alignment using RAxML v8.2.10 [41], implementing a GTR model with 1000 bootstrap replicates.

### Dating the start of the outbreak and the age of lineages

Following identification of a strong temporal signal in the 82 ST11 dataset (Fig. S4), we ran beast v2.4.7 [42] on the recombination-filtered chromosomal alignment to infer the age of the outbreak as the time to the most recent common ancestor (tMRCA). The TN93 substitution model was selected based on evaluation of all possible substitution models [43] and beast was run under different demographic model priors and allowing for both strict and relaxed models of the evolutionary rate (Table S3). In each case, the Markov chain Monte Carlo (MCMC) procedure was run for 200 million iterations and convergence of the chain was inspected using Tracer v1.6.0. The maximum clade credibility (MCC) tree under each model was generated in TreeAnnotator and plotted in ggtree [44]. The best-supported model, evaluated through path sampling, was an exponential growth model with a strict molecular clock. However, the results were very consistent across different specifications of the demographic model and mutation rate prior (Table S3, Fig. S5).

### Reconstruction of transmission chains

We applied TransPhylo [45] to reconstruct possible transmission events between the 82 outbreak isolates. We specified a gamma distribution with a shape parameter of 1 and a scaling parameter of 0.5 (6 months on average between infection and transmission) as priors on the generation time, although results were robust to variation of the scaling parameter (Figs S6 and S7). The MCMC was run for 100 000 iterations to ensure convergence, and the consensus posteriors were evaluated based on the probabilities of direct transmission between any two isolates, the average number of intermediates in the transmission chain and the number of inferred direct links (Table S4).

We also constructed epidemiological transmission matrices using hospital records, which provided information on where and when patients moved through different wards within the hospital during their admission (Fig. S2). We constructed a ‘direct-contact’ matrix where the index of transmission between any two patients was set to 1 if they were present on the same ward at the same time, and 0 otherwise. The agreement between the genetic transmission matrix and the epidemiological matrix was assessed using a Mantel test.

### *De novo* assembly and core genome phylogenies

We also assembled our Illumina short read sequence data *de novo* using SPAdes v3.10.0 [46]. The resulting assemblies were annotated using Prokka v1.12 [47], and the core and accessory genomes were defined and extracted using Roary v3.11.2 [48]. Based on the core genome alignment, we constructed a phylogeny in RAxML v8.2.10 using a GTR model and 1000 bootstrap replicates [41] (Figs S3, S8 and S9).

### AMR, virulence, plasmid and transposon profiling from WGS

*De novo* assemblies were screened against the ResFinder database of published AMR genes [49]. AMR genes were assigned using an identity threshold of 90 % and a selected minimum length of 80 %. *De novo* assemblies were also screened for genetic markers that have been associated with a virulent phenotype using the Kleborate virulence prediction tool [50]. Additionally, plasmid replicons diagnostic for incompatibility groups were identified using PlasmidFinder [51] (Tables S5-S7). The transposon structure of *bla*_KPC-2_ was elucidated through a combination of genome annotation implemented in Prokka v1.12 [47], screening for insertion sequences in ISFinder [52] and blast.

### Predicting phenotypic resistance from gene abundance

We estimated copy numbers of AMR genes by mapping our raw Illumina short-read data to our short-read *de novo* contig assemblies using Bowtie2 v2.2.6 [53]. The absolute number of reads that mapped to each gene [54], normalized by gene length and sequence depth, was used to generate a DNAseq depth of coverage, in units of transcripts per million (TPM) [55]. We tested the correlation between our generated pseudo-TPMs and phenotypic resistance (MICs) for aminoglycosides (amikacin), β-lactams (meropenem and imipenem) and quinolones (ciprofloxacin and levofloxacin). To account for the population structure in the cohort of isolates analysed, we considered each lineage separately (Figs S10–S12).

### Long-read *de novo* assembly and evaluation

PacBio long-read sequence data from eight isolates were assembled using multiple approaches. All strategies employed the *de novo* assembly pipeline implemented by UniCycler [56]. We ran Unicycler jointly using the long- and short-read data to generate a hybrid assembly, as well as also running long-read-only assemblies in both ‘normal’ and ‘bold’ modes. In addition, we ran each of these assemblies using the raw, unfiltered, PacBio reads, as well as following read quality filtering implemented in FiltLong (https://github.com/rrwick/Filtlong). Assembly quality was evaluated based on the number, depth and contiguity of the assembled contigs. The choice of assembly method selected for each isolate, together with the size and composition of the final assemblies are provided in Table S6. We inspected both the coverage and the percentage of the genome covered when mapping each of our 82 ST11 short-read sequenced isolates to each of our PacBio assemblies using bwa-mem v0.7.12 [38] (Tables S8 and S9). We found our inferred pseudo-TPMs correlated well with coverage of the relevant plasmids (Fig. S13) and showed strong correlations between genes that are known to co-occur (e.g. Fig. S10a), suggesting they provide a reliable proxy for copy number. We also evaluated the percentage identity between different assembled plasmids (Fig. S14) and annotated long-read assembled contigs to further elucidate the *bla*_KPC-2_ transposon structure (Fig. S15).

## Results

### General features of the outbreak

Following the death of a patient at PKUPH caused by a CRKP bloodstream infection in February 2016 (Patient X), a surveillance initiative was instigated to stem the subsequent outbreak and assess the presence of CRKP throughout the hospital over a 14-month period. This resulted in the identification of 100 CRKP isolates sampled from 19 wards spread over three hospital campuses, which included two intensive care unit (ICU) wards (Figs S1 and S2).

**Fig. 1.**
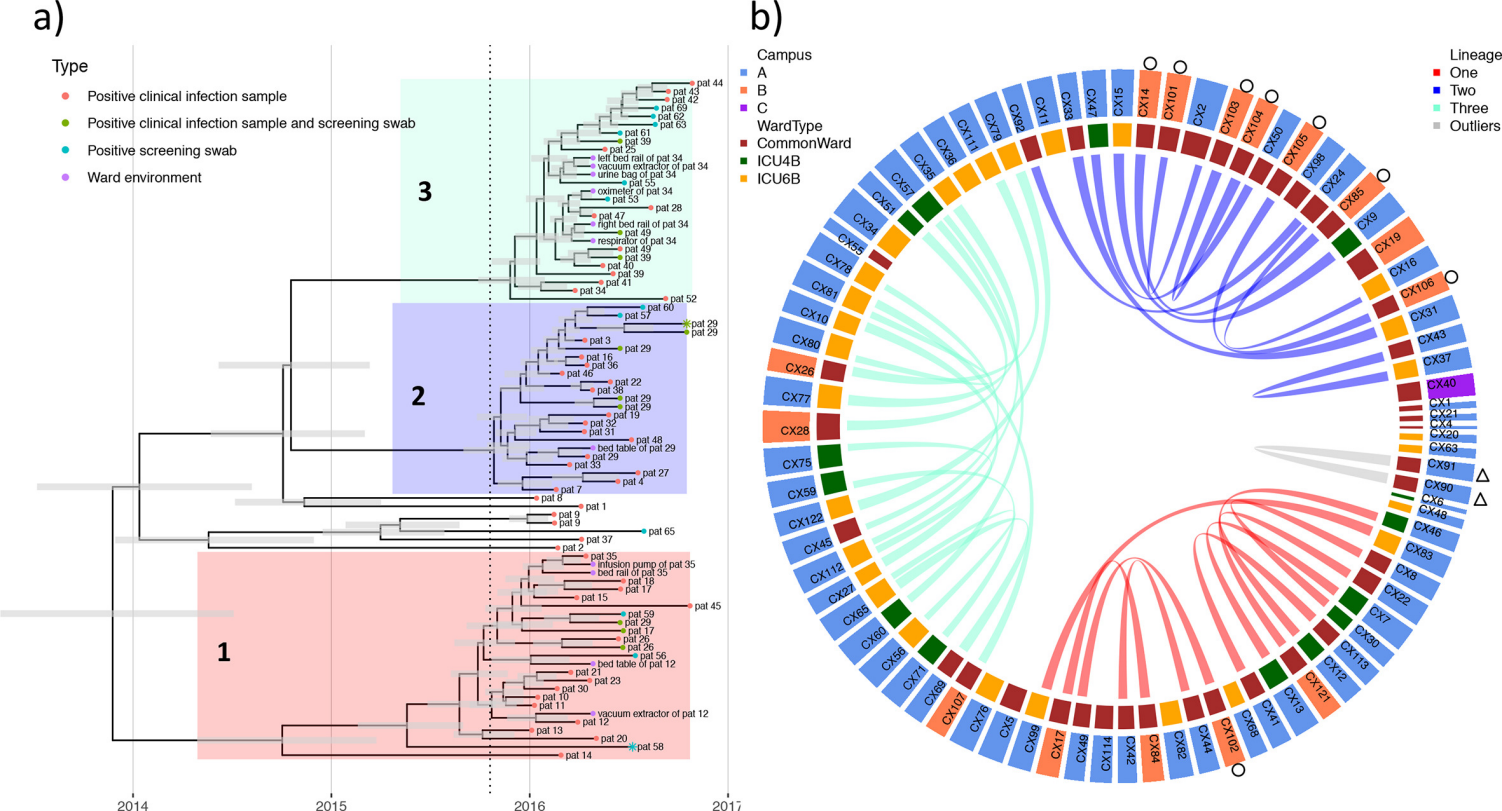
Dated phylogeny and inferred direct transmission events across outbreak isolates. (a) Dated phylogeny of the recombination-free chromosomal alignment of 82 ST11 outbreak isolates. Grey bars give the inferred 95 % highest posterior density (HPD) interval around node heights. Tips are coloured according to the infection status and isolate source. The tip symbols of two isolates are marked with an asterisk denoting the absence of *bla*_KPC-2_. The vertical dotted line provides the date the ‘index’ patient (Patient X) was first admitted. Lineages are defined as 1, 2 and 3 with seven outlying isolates. (b) Direct transmission events inferred by TransPhylo between 82 ST11 isolates. The inner ring provides the ward of isolation (either common or intensive care), while the outer ring provides the hospital campus (A, B, C), as coloured in the legends. We infer 14 direct transmissions within Lineage 3, 10 within Lineage 2 and nine within Lineage 1, with one transmission inferred between CX90 and CX91 isolated from the same patient (Patient X) 1 day apart. Isolates marked with a circle on the outer ring are from Patient 29 who was co-infected with CRKP+ strains from two lineages and was one of two sampled patients who spent time on both Campus A and Campus B during their admission. Isolates marked with a triangle were sampled from the first ‘index’ patient identified as positive for *bla*_KPC-2_, Patient X.

WGS data and phenotypic resistance profiles (MICs) were generated for all 100 CRKP isolates (Tables S1 and S2). These isolates grouped into 11 STs, of which 83/100 isolates were assigned to ST11, the dominant CRKP clone in China [23, 57] (Fig. S3). We excluded one ST11 isolate (CX3) that was a clear phylogenetic outgroup, and therefore not part of the identified outbreak. Phylogenetic analyses resolved the remaining 82 isolates into three closely related lineages (we refer to these as Lineages 1, 2 and 3) together with seven isolates falling outside of these main lineages (Fig. 1a). There was no obvious association between campus or ward of isolation and the inferred phylogenetic relationships (Fig. S8). One patient (Patient 29) was co-infected, with a single Lineage 1 isolate (CX102), and seven Lineage 2 isolates, including one isolate that was also detected from swabbing the patient’s bed table (Fig. 1a).

We found a strong temporal signal in the accumulation of mutations on the non-recombining fraction of the chromosomal sequence alignment (Fig. S4), justifying the use of tip-dating phylogenetic calibration [58]. Bayesian approaches to infer the tMRCA pointed to these isolates sharing a common ancestor in 2013 (2013.9; 95 % HPD, 2013.2–2014.4), based on an inferred evolutionary clock-rate of 3.4×10^−6^ (2.7×10^−6^ to 4.2×10^−6^) substitutions per site per year (Fig. 1a, Table S3 and Fig. S5). This inferred age pre-dates the admission of Patient X (previously considered the ‘index’ patient) by over 1 year. As such, these CRKP strains were in circulation in the hospital for around 2 years prior to initiation of the hospital-wide CRKP surveillance initiative, which only commenced after the death of Patient X.

### High transmissibility across wards and campuses

We identified 34 plausible direct links between pairs of ST11 isolates during the outbreak (Figs 1b, S6 and S7 and Table S4). Excluding links inferred from isolates sampled from the same patient, 25 direct transmissions were identified between patients, including seven within the same ward, and 18 between isolates sampled across different wards within the hospital. None of these between-patient transmissions involved either of the two isolates sampled from Patient X. Six of the between-patient transmission events occurred across hospital campuses A and B, which were located 14 km apart (Fig. S1). In our dataset, only two patients were directly transferred between campus A and B during their admissions (Patients 12 and 29). Isolates from these patients were involved in only two of the six cross-campus transmissions, suggesting a possible additional role of CRKP transfer mediated by the movement of staff or equipment.

Among the 25 direct transmissions, there were 21 instances involving at least one isolate sampled directly from one of two ICU wards. In ten of these cases, the patients involved in the direct transmission pair had overlapping admissions, which we consider as ‘direct-contacts’. In a further four cases patients were admitted to the same ICU ward within 2 weeks of each other. We found some correspondence between the transmission events inferred from the genomic data and those inferred solely from epidemiological data. There was a moderate, but statistically highly significant correlation between the matrix of transmission events inferred from the genomic data and the epidemiological ‘direct-contact’ events (*R*=0.147; *P*<0.001; Mantel test).

### Diversity in AMR gene carriage and phenotypic resistance

**Fig. 2.**
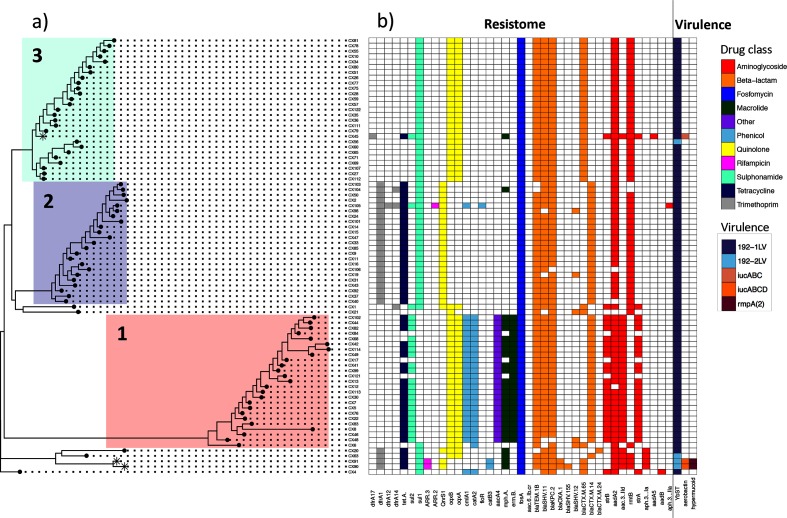
Core genome phylogeny, AMR and virulence profiles. (a) Core genome phylogeny of 82 ST11 outbreak isolates with the three-lineage structure highlighted in colour, as in Fig. 1. The three tip points marked with an asterisk (*) identify those isolates assigned as hypervirulent based on the presence of the genes *rmpA*, *rmpA2* and/or the *iuc* operon. (b) Antimicrobial resistance gene complement and inferred virulence profiles of the 82 ST11 outbreak isolates shown in (a). Coloured squares give the presence of AMR genes and genetic markers associated with virulence, with the colour of AMR gene presence providing the antibiotic drug class to which resistance is conferred, as given in the legend to the right.

The three-lineage structure was well supported by a pan-genome phylogeny (Figs 2 and S8). Despite limited variation in the core genome, we noted considerable variability in the accessory component (Fig. S9). All 82 related CRKP isolates were highly drug resistant, harbouring elements conferring resistance to aminoglycosides, β-lactams, fosfomycin, quinolones and sulphonamides, but with considerable variation in the resistance-conferring elements carried. Indeed, only one assigned resistance gene, *fosA*, was present in all 82 isolates (Fig. 2).

We identified diversity in the AMR gene component both between and within lineages. For example, CX45 in Lineage 3 carried additional resistance elements for aminoglycosides not observed in any other isolates (*dfrA17* and *aadA5*) together with resistance elements unique to its lineage, including *tetA*, *mphA*, *strA*, *strB* and *aac[3]-IId.* As well as gains, we also identified cases of AMR gene loss. For example, isolates CX103 and CX104 were unique among Lineage 2 isolates in not carrying the *rmtB* (aminoglycoside resistance) gene and *bla*_TEM-1B_. We also identified two cases of secondary loss of *bla*_KPC-2_ in CX48 and CX106 (Figs 1a and 2). Although these may represent losses in culture, they could also plausibly represent genuine secondary losses in patients.

As well as AMR gene presence and absence, CRKP isolates showed considerable variation in phenotypic resistance to different drug classes (Table S2). Isolates from closely related lineages often had different resistance mechanisms present, including mobile genes associated with the accessory genome. Resistance gene abundances, as measured through pseudo-TPMs (see Methods), could in some cases be quantitatively associated with AMR phenotype, measured by MICs. We identified lineages with a significant correlation between gene abundance and resistance phenotype response for aminoglycosides (Fig. S10), β-lactams (Fig. S11) and quinolones (Fig. S12). This suggests that gene copy number variation, for example mediated through the carriage of multiple plasmids carrying the same AMR gene, beyond simple presence and absence, may contribute to AMR phenotypes in a quantitatively predictive way.

### Diversity in virulence genes

We screened for genes associated with increased pathogenicity [50]. All isolates carried the yersiniabactin siderophore (type *ybt9*) on an integrative conjugative element (ICEKp3), suggesting a common mechanism for acquiring iron to support growth in host tissues (Fig. 2). Three isolates also carried the acquired aerobactin siderophore gene (*iuc*), which is involved in scavenging iron from host blood transferrin [59] and is a commonly used genetic marker of virulence. Two of these isolates were from Patient X (CX90, CX91) and one was from Patient 55 (CX45). CX90 and CX91 also carried the genes *rmpA* and *rmpA2*, which are associated with both virulence and a hypermucoviscous phenotype [50, 60, 61]. While Patient X died as a result of CRKP infection, Patient 55 recovered and was discharged.

### Diversity in plasmid carriage

We assigned plasmid incompatibility groups to explore carriage of the identified AMR and virulence determinants. Plasmid carriage was high, with 3.74 (mean) unique matching plasmid replicons identified per isolate. Amongst these, all isolates showed evidence of carrying an IncFII-like plasmid, with all but one (CX68) also carrying an ~10 060 bp (ColRNAI) plasmid (Table S5). Lineage 1 and Lineage 2 isolates carried on average more plasmids (based on identified replicons) than Lineage 3 (mean per isolate: 4.84, 4.22, 2.22, respectively). Despite the homology of the chromosomes of Lineage 2 and Lineage 3 (pairwise SNP difference across lineages of 42–57 (95 % confidence interval [CI], Fig. S4a), their plasmid carriage was strikingly different.

**Fig. 3.**
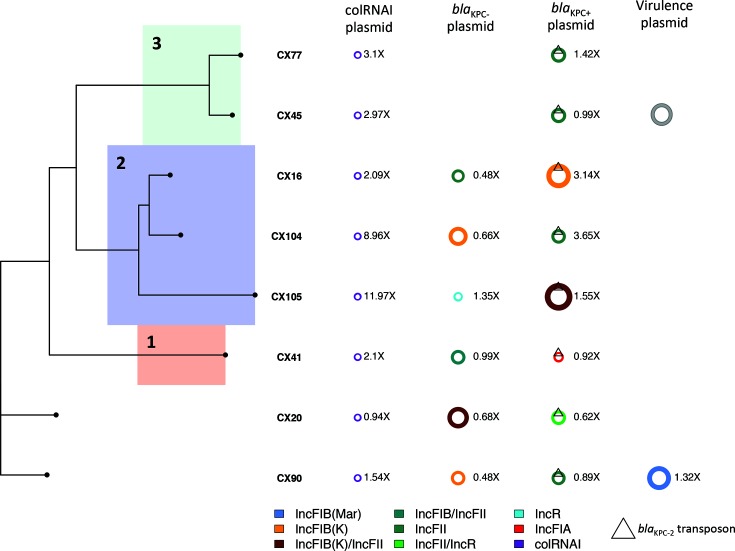
Diversity in plasmid type and carriage across eight isolates involved in the outbreak. Complement of plasmids carried by eight isolates assembled using PacBio long-read technology, sampled over the time-course of the outbreak. The colour provides the Inc type assignment of the plasmids based on the presence of plasmid replicon sequences and is given in the legend at the bottom. Plasmid copy number relative to coverage over the chromosome is provided to the right of each depicted plasmid. *bla*_KPC-2_-carrying plasmids are denoted with the triangular symbol and are found in the third column. Virulence plasmids are defined as those carrying the *rmpA*, *rmpA2* and/or *iuc* operon genes. We have also included the virulence plasmid of CX45 (in grey), which we were able to identify from short-read sequencing. The phylogeny on the left provides the core genome phylogeny for these eight isolates, with chromosomal Lineages 1–3 highlighted in colour, as in Figs 1 and 2.

We explored the accessory genomes of CRKP isolates further using long-read sequencing assemblies of eight isolates from the outbreak cohort (Tables S6 and S7, Fig. 3). Consistent with our assignments based on identified plasmid replicons, Lineage 3 assemblies carried one fewer plasmid. Although short-read assemblies and genetic markers of resistance and virulence suggested the presence of an additional plasmid carried uniquely in Lineage 3 (isolate CX45), we were unable to identify this using long-read sequencing, suggesting this plasmid may have been lost in the culture for long-read sequencing of isolate CX45 (Fig. 3).

Long-read assemblies allowed us to observe multiple gains and losses of plasmids, leading to extensive variations in plasmid component and copy number across the 82 ST11 isolates (Fig. 3, Tables S6, S8 and S9). For example, the ColRNAI plasmid was often present at high copy relative to the chromosome (0.94–11.97 95 % CI). We also noted differences in coverage of the *bla*_KPC-2_-carrying plasmid, with Lineage 2 isolates tending to carry *bla*_KPC-2_ plasmids at higher copy number (Table S8). This is in good agreement with our analysis of the correlation between β-lactam gene copy number and quantitative phenotypic resistance, which identified *bla*_KPC-2_ as a key driver of β-lactam resistance in Lineage 2 (Fig. S11b,c). We also identified at least one instance of large genomic regions shared between plasmids, consistent with putative recombination between them (Fig. S14).

**Fig. 4.**
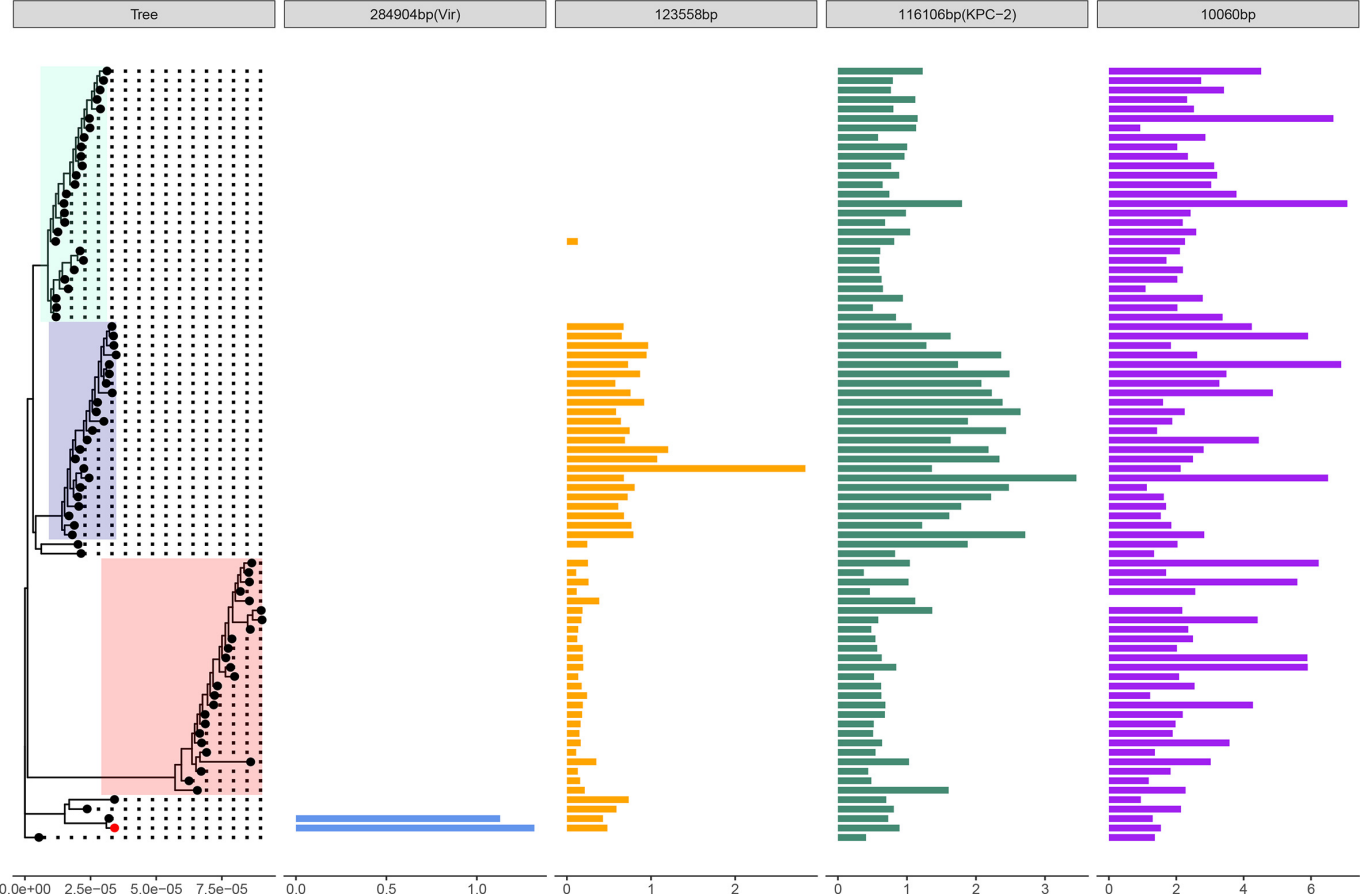
Coverage of outbreak isolates to the CX90 assembly highlights variation in plasmid carriage and copy number. Mean coverage per isolate of 82 short-read sequenced ST11 *K. pneumoniae* mapped to the complete genome of CX90 (red tip point), sampled from the ‘index’ patient, Patient X, and sequenced using long-read technology. The phylogeny to the left provides the chromosomally dated phylogeny of 82 outbreak isolates, with the lineage structure highlighted in colour, as in Figs 1–3. The bar plots provide the mean coverage relative to the chromosome for each plasmid. Any isolates with a mean coverage relative to the chromosome <0.1× are not shown.

Isolate CX90 from Patient X had an additional large IncFIB (Mar) *bla*_OXA-1_-positive (284 904 bp) plasmid which had high sequence similarity to a previously sequenced 288 222 bp virulence plasmid isolated from a human host at Chengdu, Sichuan, in July 2016 (CP028791.1). As well as carrying AMR genes involved in fluoroquinolone and aminoglycoside resistance (*aac(6')Ib-cr*), phenicol resistance (*catB3*), rifampicin resistance (*arr-3*) and sulphonamide resistance (*sul1*), this plasmid carried the previously identified *rmpA* and *rmpA2* markers of hypermucovisity and *iucABCD* operon involved in aerobactin synthesis (Fig. 2). This plasmid was absent from all isolates other than those from Patient X. Because these two highly related isolates were not involved in further transmissions to other patients (Fig. 1a), it is likely that this plasmid was acquired *de novo* by the strain infecting Patient X (Fig. 4, Tables S8 and S9).

### High mobility of *bla*_KPC-2_

*bla*_KPC-2_ was found on 80 conserved contigs in the ST11 outbreak cohort, ranging in size from 2393 to 9266 bp. All *bla*_KPC-2_ were found on TN1722-based isoforms with a transposon structure of type ΔISKpn6-*KPC-2*-ΔISKpn27-ΔTnpR (Fig. S15) and contained two promoters associated with the *bla*_KPC-2_ transcription start sites: the intrinsic promoter P1 and the upstream promoter P2 [62].

Notably, based on our long-read assemblies, *bla*_KPC-2_ was found on a variety of different IncF plasmid backgrounds: IncFII (*n*=4), IncFII also carrying an IncR-associated replicon (*n*=1), IncFIA (*n*=1), IncFIB(K) (*n*=1) and IncFIB(K) with an IncFII-associated replicon (*n*=1) (Fig. 3, Table S7, Fig. S15). The diversity of *bla*_KPC-2_ plasmid backgrounds is best explained by frequent transpositions and acquisitions of *bla*_KPC-2_ between plasmids during the course of the outbreak.

## Discussion

In this work we identified an outbreak of 82 closely related ST11 *bla*_KPC-2_-positive isolates during a 14-month surveillance initiative at a Beijing hospital. Using WGS analyses, we show that these isolates had been in circulation for at least 1 year prior to the initiation of our sampling efforts and had accumulated extensive genetic and phenotypic diversity that cannot be captured by commonly used typing schemes which rely on variation on the chromosome.

The application of WGS allowed us to gain deeper insight into the extensive transmission of these isolates between patients. For example, the patient initially suspected to be the index case (Patient X) was probably not involved in any subsequent transmission to other patients. Interestingly, the two CRKP samples isolated from Patient X were unique in their carriage of a *bla*_OXA-1_ virulence plasmid, probably acquired by the infecting strain, which may have contributed to the fatal outcome of this patient.

Our reconstruction of transmission events during the outbreak suggested that ICUs were a key environment for direct patient-to-patient transmission. Whilst in many instances these transmissions correlated well with the movement of patients through the hospital based on admission records, we also inferred transmissions within our dataset (e.g. across hospital campuses) which could not be predicted by the epidemiological data alone. This suggests a possible role of the movement of staff, equipment or further unsampled sources in the transmission of nosocomial pathogens, as well as highlighting the need to integrate both genetic and epidemiological data to fully track and reconstruct outbreaks.

Despite all 82 isolates having closely related chromosomes (a mean of 58 pairwise SNP differences across all lineages; 95 % CI 8–96 SNPs), we identified marked accessory genome diversity, including variation in resistance genes, virulence-associated genes and plasmid carriage. For example, we were able to identify a plasmid carried uniquely in a single patient, between-plasmid recombination events and transposon-mediated exchange of the *bla*_KPC-2_ gene. Remarkably, only a single recognized AMR gene was present in all 82 isolates (*fosA*). This highlights how plasticity in the accessory genome occurs even at small geographical and temporal scales. We also identified examples where plasmid copy number variation correlated with quantitative AMR phenotypes, indicating that the relative abundance and stability of AMR-carrying plasmids may be an important and largely uncharacterized contributor to levels of phenotypic resistance in hospital strains. Exploring the nature of this relationship in other closely related drug-resistant lineages, and for other antibiotic classes, is an important area of future work.

High mobility of *bla*_KPC_ elements has been previously documented in a Tn*4401b bla*_KPC_-carrying cohort from a tertiary care hospital in Virginia, USA [16], and during long-term colonization of a patient in the USA [32]. The analysis of this outbreak further confirms the extraordinary potential for mobility of *bla*_KPC-2_-carrying transposons. Indeed, the *bla*_KPC-2_ element was found on four different plasmid backgrounds in the eight outbreak strains that were sequenced with long reads. Intriguingly, given the extraordinary rate of transposition of the *bla*_KPC-2_-carrying transposon and the rapid turnover of the plasmid complement, we did not identify a single outbreak strain carrying more than one copy of *bla*_KPC-2_.

Our findings further confirm that CRKP represents a significant AMR threat [5] requiring increased global study and surveillance. The fast genotypic evolution that we describe in *K. pneumoniae* lineages, through multiple gains, losses and rearrangement of the accessory genome, probably allows this nosocomial pathogen to rapidly adapt to essentially any antibiotic used in treatment over very short periods of time.

The use of WGS is becoming widespread in clinical microbiology, but analysis often relies on reference-based mapping, which focuses on genetic variation on the chromosome, which remained relatively conserved in these outbreak lineages and did not well recapitulate either the dynamics of the accessory genome or the resistance phenotype. This highlights the need for better WGS-based diagnostics tools which incorporate plasmid characterization, such as resistance gene copy number variation, to tackle the growing incidence of multi-drug-resistant nosocomial infections and provide patients with the best care possible in the future.

## Data Bibliography

1. Raw Illumina short-read and PacBio long-read sequences have been deposited on NCBI with BioProject IDPRJNA508760. Full accompanying metadata and phenotypic resistance characterization are available in Tables S1 and S2.

## Supplementary Data

Supplementary File 1
