# Supplementary material for: Rapid phenotypic evolution in multidrug-resistant Klebsiella pneumoniae hospital outbreak strains
Source: Microb Genom. 2019 Apr 2;5(4):e000263. doi: 10.1099/mgen.0.000263 (PMC6521586; doi:10.1099/mgen.0.000263)
Supplement: Supplementary File 1 [file mgen-5-263-s001.pdf]

Supplementary Figures and Tables

Rapid phenotypic evolution in multidrug resistant *Klebsiella pneumoniae* hospital outbreak strains

Contents

S1 Dataset collection, CRKP surveillance and intervention strategies 3

S2 Inferring a timed phylogeny and transmission networks 12

S2.1 BEAST analyses . . . 12

S2.2 TransPhylo analyses . . . 14

S3 Inferring core and accessory genome structure 17

S4 AMR gene copy number variation (CNV) and phenotypic resistance 19

S5 Plasmid assemblies 22

S5.1 Plasmid copy number variation . . . 23

S5.2 Accessory genome mobility . . . 25

S5.3 bla_{KPC} mobility . . . 26

List of Figures

S1 The hospital ward location map of all infected patients sampled during CRKP surveillance. 3

S2 Patient admission information, including movement through hospital wards and time points of sampling for CRKP and WGS. . . . 10

S3 Core genome maximum likelihood phylogeny of 100 isolates sampled as part of the CRKP surveillance initiative. . . . 11

S4 Temporal signal across 82 ST11 outbreak isolates. . . . 12

S5 Posterior distribution around inferred tMRCA under 6 alternate BEAST models . . . 13

S6 TransPhylo consensus transmission tree . . . 14

S7 TransPhylo scale parameter sensitivity analysis . . . 15

S8 Core genome phylogeny of 82 ST11 outbreak isolates . . . 17

S9 Core and accessory gene tree . . . 18

S10 Associations between aminoglycoside resistance genes and phenotypic resistance to the aminoglycoside antibiotic amikacin. . . . 19

S11 Associations between beta-lactam resistance genes and phenotypic resistance to beta-lactam antibiotics. . . . 20

S12 Associations between quinolone resistance genes and phenotypic resistance to quinolone antibiotics . . . 21

S13 Relationship between AMR gene pseudo-TPMs and plasmid coverage. . . . 24

S14 Blast identity (%) between two plasmids assembled using long-read data from CX20 . 25

S15 Genomic background and $bla_{\mathrm{KPC+}}$ TN*1722* type transposon structure. . . . 26

List of Tables

S1 Description of full dataset . . . 4
S2 Phenotypic resistance (MICs) for full dataset . . . 7
S3 Inferred tMRCA, clock rates and marginal likelihoods inferred by Bayesian dating analyses 13
S4 TransPhylo inferred direct transmission pairings . . . 16
S5 Presence of plasmid replicons across isolates . . . 18
S6 PacBio assembly statistics . . . 22
S7 Presence of plasmid replicons across PacBio long-read assemblies . . . 22
S8 Copy number variation (CNV) amongst assembled plasmids . . . 23
S9 Percentage of the genome covered amongst assembled plasmids . . . 23

S1 Dataset collection, CRKP surveillance and intervention strategies

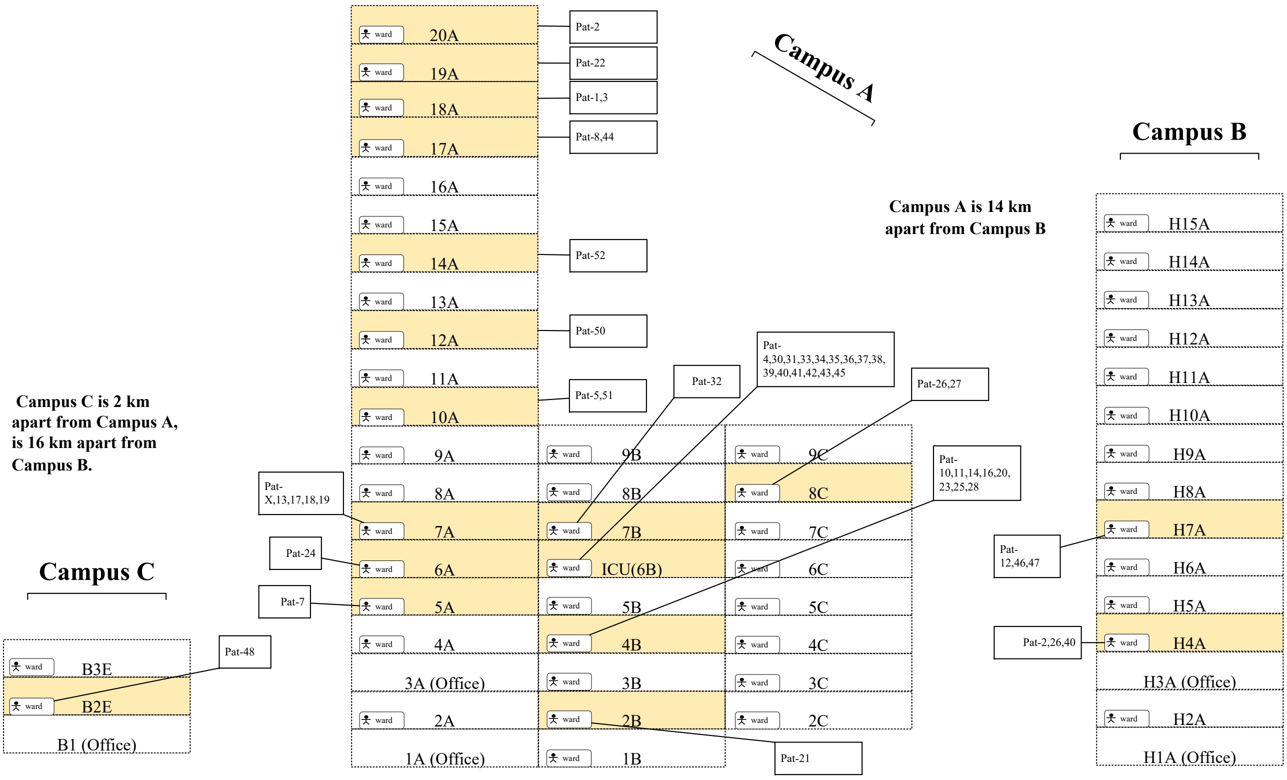

Figure S1: Schematic representation of the PKUPH hospital and ward locations across campuses A, B and C. Wards are coloured yellow to indicate where CRKP+ isolates were identified with labels providing the patients included in the final dataset. Full details of the final collection are provided in Table S1.

Table S1: The full meta-data for isolates collected from patients over the course of the CRKP surveillance period. Short read sequence IDs marked with a '*' were also sequenced using PacBio long read technology. The lineage assignments (Lin.) and MLST groupings are provided based on bioinformatic analyses.

Patient	ID	Type	MLST	Date	Specimen	Ward	Category	Department	Campus	Disease	Lin.	Outcome
6	CX3	infection	11	30/07/2015	sputum	H4A	Common	ICU	B	Acute Myeloid Leukemia-M4	O	Cured
5	CX94	infection	307	23/10/2015	sputum	10A	Common	Hematology	A	Acute Myeloid Leukemia	O	Cured
5	CX95	infection	307	07/11/2015	sputum	10A	Common	Hematology	A	Acute Myeloid Leukemia	O	Cured
5	CX96	infection	307	14/11/2015	sputum	10A	Common	Hematology	A	Acute Myeloid Leukemia	O	Cured
5	CX97	infection	307	01/12/2015	sputum	10A	Common	Hematology	A	Acute Myeloid Leukemia	O	Cured
13	CX8	infection	11	04/01/2016	pertioneal	7A	Common	Hepatobiliary	A	Obstructed Jaundice	1	Abandon
11	CX13	infection	11	09/01/2016	sputum	ICU(4B)	ICU(4B)	ICU	A	Liver cancer	1	Abandon
8	CX21	infection	11	13/01/2016	urine	17A	Common	Geriatrics	A	Coronary heart disease	O	Cured
10	CX7	infection	11	15/01/2016	pertioneal	ICU(4B)	ICU(4B)	ICU	A	Liver transplantation	1	Cured
X	CX91	infection	11	14/02/2016	liver abscess	7A	Common	Hepatobiliary	A	Gallbladder carcinoma	O	Die
X	CX90*	infection	11	15/02/2016	blood	7A	Common	Hepatobiliary	A	Gallbladder carcinoma	O	Die
7	CX98	infection	11	18/02/2016	sputum	5A	Common	Cardiology	A	Pulmonary infection	2	Cured
30	CX76	infection	11	20/02/2016	catheter	ICU(6B)	ICU(6B)	ICU	A	Pulmonary infection	1	Cured
2	CX4	infection	11	21/02/2016	sputum	20A	Common	Hematology	A	Acute Myeloid Leukemia-M5	O	Cured
14	CX6	infection	11	27/02/2016	blood	ICU(4B)	ICU(4B)	ICU	A	Liver cirrhosis	1	Die
46	CX19	infection	11	29/02/2016	sputum	H7A	Common	Comprehensive	B	Pulmonary infection	2	Die
33	CX11	infection	11	14/03/2016	trachea secretion	ICU(6B)	ICU(6B)	ICU	A	Pulmonary infection	2	Die
21	CX22	infection	11	16/03/2016	cervicovaginal	2B	Common	Obstetrics	A	Gestation	1	Cured
34	CX10	infection	11	24/03/2016	urine	ICU(6B)	ICU(6B)	ICU	A	Cerebral hemorrhage	3	Die
15	CX5	infection	11	27/03/2016	blood	7A	Common	Hepatobiliary	A	Liver transplantation	1	Cured
12	CX17	infection	11	29/03/2016	sputum	H7A	Common	Comprehensive	B	Pulmonary infection	1	Cured
1	CX1	infection	11	04/04/2016	blood	18A	Common	Hematology	A	Acute Myeloid Leukemia-M4	O	Die
16	CX9	infection	11	05/04/2016	blood	ICU(4B)	ICU(4B)	ICU	A	Liver cancer	2	Die
37	CX20*	infection	11	05/04/2016	sputum	ICU(6B)	ICU(6B)	ICU	A	Respiratory failure	O	Cured
31	CX16*	infection	11	10/04/2016	secretion	ICU(6B)	ICU(6B)	ICU	A	Respiratory failure	2	Cured
3	CX2	infection	11	11/04/2016	blood	18A	Common	Hematology	A	Acute Lymphocytic Leukemia	2	Die
32	CX24	infection	11	12/04/2016	blood	7B	Common	Gastroenterology	A	Liver cirrhosis	2	Die
35	CX99	infection	11	13/04/2016	urine	ICU(6B)	ICU(6B)	ICU	A	Respiratory failure	1	Die
36	CX15	infection	11	15/04/2016	blood	ICU(6B)	ICU(6B)	ICU	A	Acute Myeloid Leukemia-M5	2	Die
29	CX14	infection	11	16/04/2016	urine	H4A	Common	ICU	B	Cerebral infarction	2	Abandon
23	CX12	infection	11	20/04/2016	sputum	ICU(4B)	ICU(4B)	ICU	A	Multiple fracture	1	Abandon
38	CX31	infection	11	25/04/2016	sputum	ICU(6B)	ICU(6B)	ICU	A	Acute pancreatitis	2	Die
oximeter 34	CX77*	environmental	11	26/04/2016	environmental	ICU(6B)	ICU(6B)	ICU	A	Cerebral hemorrhage	3	Die
bed rail 35	CX82	environmental	11	26/04/2016	environmental	ICU(6B)	ICU(6B)	ICU	A	Respiratory failure	1	Die
vacuum extractor 12	CX84	environmental	11	26/04/2016	environmental	H7A	Common	Comprehensive	B	Pulmonary infection	1	Cured
bed table 29	CX85	environmental	11	26/04/2016	environmental	H4A	Common	ICU	B	Cerebral infarction	2	Abandon
respirator 34	CX80	environmental	11	26/04/2016	environmental	ICU(6B)	ICU(6B)	ICU	A	Cerebral hemorrhage	3	Die
right bed rail 34	CX81	environmental	11	26/04/2016	environmental	ICU(6B)	ICU(6B)	ICU	A	Cerebral hemorrhage	3	Die
vacuum extractor 34	CX122	environmental	11	26/04/2016	environmental	ICU(6B)	ICU(6B)	ICU	A	Cerebral hemorrhage	3	Die

. . . continued on next page. . .

Patient	ID	Type	MLST	Date	Specimen	Ward	Ward Category	Department	Campus	Disease	Lin.	Outcome
urine bag 34	CX78	environmental	11	26/04/2016	environmental	ICU(6B)	ICU(6B)	ICU	A	Cerebral hemorrhage	3	Die
left bed rail 34	CX79	environmental	11	26/04/2016	environmental	ICU(6B)	ICU(6B)	ICU	A	Cerebral hemorrhage	3	Die
infusion pump 35	CX83	environmental	11	26/04/2016	environmental	ICU(6B)	ICU(6B)	ICU	A	Respiratory failure	1	Die
bed table 12	CX121	environmental	11	26/04/2016	environmental	H7A	Common	Comprehensive	B	Pulmonary infection	1	Cured
47	CX28	infection	11	28/04/2016	pertioneal	H7A	Common	Comprehensive	B	Gallbladder carcinoma	3	Die
20	CX30	infection	11	02/05/2016	sputum	ICU(4B)	ICU(4B)	ICU	A	Pulmonary infection	1	Cured
41	CX27	infection	11	11/05/2016	sputum	ICU(6B)	ICU(6B)	ICU	A	Respiratory failure	3	Cured
40	CX34	infection	11	14/05/2016	blood	ICU(6B)	ICU(6B)	ICU	A	Guillain-Barre syndrome	3	Die
24	CX29	infection	1905	17/05/2016	wound	6A	Common	Gastrointestinal	A	Gastrointestinal hemorrhage	O	Cured
25	CX75	infection	11	18/05/2016	sputum	ICU(4B)	ICU(4B)	ICU	A	Bladder cancer	3	Die
53	CX35	+ve CRKP screen	11	23/05/2016	urethral swabs	ICU(6B)	ICU(6B)	ICU	A	Thrombocytopenic purpura	3	Cured
19	CX92	infection	11	25/05/2016	sputum	7A	Common	Hepatobiliary	A	Duodenal ulcer	2	Abandon
22	CX33	infection	11	28/05/2016	sputum	19A	Common	Hepatobiliary	A	Bone Tumors	2	Die
39	CX36	infection	11	02/06/2016	urine	ICU(6B)	ICU(6B)	ICU	A	Coronary heart disease	3	Cured
54	CX38	+ve CRKP screen	307	09/06/2016	throat	ICU(6B)	ICU(6B)	ICU	A	Guillain-Barre syndrome	O	Abandon
4	CX37	infection	11	11/06/2016	blood	ICU(6B)	ICU(6B)	ICU	A	Acute Lymphocytic Leukemia	2	Die
26	CX41*	infection	11	12/06/2016	sputum	8C	Common	Neurosurgery	A	Cerebral hemorrhage	1	Cured
49	CX26	infection	11	15/06/2016	axilla	H4A	Common	ICU	B	Pulmonary infection	3	Die
57	CX47	+ve CRKP screen	11	15/06/2016	rectal swab	ICU(4B)	ICU(4B)	ICU	A	Cerebral hernia	2	Cured
61	CX57	+ve CRKP screen	11	15/06/2016	rectal swab	ICU(4B)	ICU(4B)	ICU	A	Cerebral hemorrhage	3	Die
29	CX101	+ve screen & clinical	11	15/06/2016	rectal swab	H4A	Common	ICU	B	Cerebral infarction	2	Abandon
29	CX102	+ve screen & clinical	11	15/06/2016	axilla	H4A	Common	ICU	B	Cerebral infarction	1	Abandon
29	CX103	+ve screen & clinical	11	15/06/2016	nose	H4A	Common	ICU	B	Cerebral infarction	2	Abandon
29	CX104*	+ve screen & clinical	11	15/06/2016	throat	H4A	Common	ICU	B	Cerebral infarction	2	Abandon
49	CX107	+ve screen & clinical	11	15/06/2016	axilla	H4A	Common	ICU	B	Pulmonary infection	3	Die
39	CX111	+ve screen & clinical	11	15/06/2016	rectal swab	ICU(6B)	ICU(6B)	ICU	A	Coronary heart disease	3	Cured
39	CX112	+ve screen & clinical	11	15/06/2016	rectal swab	ICU(6B)	ICU(6B)	ICU	A	Coronary heart disease	3	Cured
17	CX42	infection	11	16/06/2016	urine	7A	Common	Hepatobiliary	A	Liver cancer	1	Cured
26	CX113	+ve screen & clinical	11	20/06/2016	nose	8C	Common	Neurosurgery	A	Cerebral hemorrhage	1	Cured
18	CX44	infection	11	21/06/2016	rectal swab	7A	Common	Hepatobiliary	A	Gaslrointestinal perforation	1	Cured
59	CX49	+ve CRKP screen	11	21/06/2016	rectal swab	7A	Common	Hepatobiliary	A	Obstructed Jaundice	1	Cured
17	CX114	+ve screen & clinical	11	21/06/2016	rectal swab	7A	Common	Hepatobiliary	A	Liver cancer	1	Cured
55	CX45*	+ve CRKP screen	11	23/06/2016	rectal swab	7A	Common	Hepatobiliary	A	Gastric carcinoma	3	Cured
18	CX116	+ve screen & clinical	1129-2LV	24/06/2016	rectal swab	7A	Common	Hepatobiliary	A	Gastrointestinal perforation	O	Cured
24	CX109	+ve screen & clinical	161-1LV	27/06/2016	rectal swab	6A	Common	Gastrointestinal	A	Gastrointestinal hemorrhage	O	Cured
24	CX110	+ve screen & clinical	161-1LV	27/06/2016	throat	6A	Common	Gastrointestinal	A	Gastrointestinal hemorrhage	O	Cured
48	CX40	infection	11	06/07/2016	blood	B2E	Common	Liver Disease	C	Liver cirrhosis	2	Abandon
58	CX48	+ve CRKP screen	11	08/07/2016	rectal swab	ICU(6B)	ICU(6B)	ICU	A	Cerebral infarction	1	Cured
56	CX46	+ve CRKP screen	11	13/07/2016	rectal swab	ICU(4B)	ICU(4B)	ICU	A	Femoral fracture	1	Cured
27	CX43	infection	11	18/07/2016	urine	8C	Common	Neurosurgery	A	Cerebral hemorrhage	2	Die
60	CX50	+ve CRKP screen	11	27/07/2016	rectal swab	8C	Common	Neurosurgery	A	Subdural edema	2	Abandon
27	CX115	+ve screen & clinical	416-1LV	27/07/2016	rectal swab	8C	Common	Neurosurgery	A	Cerebral hemorrhage	O	Die
66	CX66	+ve CRKP screen	370	28/07/2016	rectal swab	ICU(6B)	ICU(6B)	ICU	A	Pulmonary infection	O	Cured
65	CX63	+ve CRKP screen	11	29/07/2016	rectal swab	ICU(6B)	ICU(6B)	ICU	A	Respiratory failure	O	Cured

. . . continued on next page. . .

Patient	ID	Type	MLST	Date	Specimen	Ward	Ward Category	Department	Campus	Disease	Lin.	Outcome
64	CX61	+ve CRKP screen	26-1LV	04/08/2016	rectal swab	ICU(4B)	ICU(4B)	ICU	A	Pancreatic carcinoma	O	Cured
68	CX70	+ve CRKP screen	656	04/08/2016	rectal swab	ICU(4B)	ICU(4B)	ICU	A	Breast Cancer	O	Die
28	CX51	infection	11	11/08/2016	sputum	ICU(4B)	ICU(4B)	ICU	A	Rectal carcinoma	3	Die
62	CX59	+ve CRKP screen	11	15/08/2016	rectal swab	ICU(4B)	ICU(4B)	ICU	A	Endometrial hyperplasia	3	Cured
63	CX60	+ve CRKP screen	11	19/08/2016	rectal swab	ICU(4B)	ICU(4B)	ICU	A	Ovarian carcinoma	3	Die
69	CX71	+ve CRKP screen	11	21/08/2016	rectal swab	ICU(4B)	ICU(4B)	ICU	A	Colon Cancer	3	Cured
67	CX67	+ve CRKP screen	15	23/08/2016	rectal swab	ICU(4B)	ICU(4B)	ICU	A	Lung carcinoma	O	Cured
52	CX55	infection	11	07/09/2016	bone marrow	14A	Common	Rheumatology	A	Fever of unknown origin	3	Cured
42	CX56	infection	11	10/09/2016	blood	ICU(6B)	ICU(6B)	ICU	A	Respiratory failure	3	Die
43	CX65	infection	11	12/09/2016	trachea secretion	ICU(6B)	ICU(6B)	ICU	A	Invasive Fungal Disease	3	Abandon
70	CX73	+ve CRKP screen	182	23/09/2016	rectal swab	H15A	Common	Hematology	B	Acute Myeloid Leukemia-M5	O	Cured
50	CX58	infection	2535	24/09/2016	urine	12A	Common	Orthopedics	A	Periprosthetic joint infection	O	Cured
29	CX105*	+ve screen & clinical	11	16/10/2016	axilla	H4A	Common	ICU	B	Cerebral infarction	2	Abandon
29	CX106	+ve screen & clinical	11	16/10/2016	throat	H4A	Common	ICU	B	Cerebral infarction	2	Abandon
51	CX72	infection	629	19/10/2016	urine	10A	Common	Hematology	A	Acute Myeloid Leukemia	O	Cured
45	CX68	infection	11	22/10/2016	blood	ICU(6B)	ICU(6B)	ICU	A	Liver transplantation	1	Abandon
44	CX69	infection	11	26/10/2016	urine	17A	Common	Geriatrics	A	Viral hepatitis	3	Cured

Table S2: The phenotypic resistance profiles, measured through MICs, for isolates collected over the course of the CRKP surveillance period. Entries marked 'ND' were not tested.

ID	meropenem	imipenem	polymyxin	tigecycline	cefoxitin	cefepime	ceftazidime	cefotaxime	ceftriaxone	cefperazone	piperacillin	amikacin	ciprofloxacin	levofloxacin	minocyline	fosfomycin
CX1	>32	16	0.25	0.5	128	64	64	>256	256	>256	>256	>256	>64	64	1	>256
CX10	>32	16	0.25	0.5	128	64	32	256	>256	256	>256	>256	64	32	1	>256
CX101	>32	16	0.5	4	>256	128	64	128	128	>256	>256	>256	>64	>64	32	>256
CX102	>32	>32	0.5	4	>256	256	256	256	>256	>256	>256	1	>64	>64	64	>256
CX103	>32	16	0.5	1	256	64	32	64	128	128	256	1	64	32	4	>256
CX104	>32	16	0.5	1	256	64	64	128	256	>256	256	1	64	32	32	>256
CX105	>32	16	0.5	4	>256	64	32	128	128	>256	>256	>256	>64	>64	32	>256
CX106	0.25	0.125	0.5	4	256	16	2	32	64	64	16	>256	>64	>64	8	>256
CX107	>32	16	0.5	0.5	256	64	16	256	>256	256	>256	>256	64	32	2	>256
CX109	>32	>32	0.5	1	128	128	64	256	>256	>256	>256	1	32	16	4	256
CX11	>32	>32	0.25	1	>256	128	128	128	256	>256	>256	>256	32	32	8	>256
CX110	>32	32	0.5	1	128	128	64	256	>256	>256	>256	1	32	16	4	256
CX111	>32	16	0.5	0.5	128	64	32	>256	>256	>256	>256	>256	64	32	1	>256
CX112	>32	16	0.5	0.5	128	64	32	>256	>256	>256	>256	>256	64	32	1	>256
CX113	>32	32	0.5	4	256	128	64	128	256	>256	>256	1	>64	>64	128	>256
CX114	>32	>32	0.5	4	256	64	32	128	256	256	>256	2	>64	>64	128	>256
CX115	32	8	0.5	1	256	32	32	64	128	>256	>256	>256	64	32	8	>256
CX116	>32	>32	0.5	4	>256	128	128	256	>256	>256	>256	1	>64	>64	64	>256
CX12	>32	>32	0.125	2	256	128	32	128	256	256	>256	>1024	>64	>64	128	ND
CX121	>32	>32	0.5	2	256	64	64	128	256	256	>256	2	64	16	2	>256
CX122	>32	16	0.5	0.25	256	64	32	>256	>256	>256	>256	>256	64	32	1	>256
CX13	>32	>32	0.25	4	256	64	32	128	256	256	>256	2	>64	>64	64	>256
CX14	>32	16	0.5	4	256	64	32	128	256	>256	>256	>256	>64	>64	32	>256
CX15	>32	16	0.5	1	256	64	32	128	256	>256	>256	>256	32	32	8	>256
CX16	>32	16	32	2	128	64	32	128	256	>256	>256	>256	>64	>64	16	>256
CX17	>32	32	0.25	0.5	128	64	128	128	256	>256	>256	2	32	16	1	>256
CX19	>32	8	0.25	1	256	64	32	128	256	>256	>256	>256	32	32	8	>256
CX2	>32	16	0.125	1	256	128	64	128	256	>256	>256	>256	32	32	32	>256
CX20	>32	16	0.25	2	128	64	128	256	>256	256	>256	1	32	32	4	>256
CX21	16	4	0.25	0.5	128	64	>256	256	>256	256	256	>256	32	16	4	256
CX22	>32	>32	0.25	4	>256	128	64	>256	>256	>256	>256	1	>64	64	128	>256
CX24	32	8	0.25	1	256	64	32	64	128	>256	>256	>256	32	32	8	>256
CX26	32	8	0.25	0.5	256	64	32	256	>256	256	>256	>256	64	32	2	256
CX27	>32	>32	0.25	0.25	128	64	32	>256	>256	256	>256	>1024	64	32	2	ND
CX28	32	8	0.25	0.5	128	32	256	128	128	256	>256	>256	>64	32	4	>256
CX29	2	4	0.25	2	16	16	64	32	128	128	>256	0.5	32	16	8	16
CX3	4	32	0.25	2	>256	32	64	64	16	16	>256	32	>64	>64	16	256
CX30	>32	>32	0.125	2	256	64	32	128	256	256	>256	1	>64	>64	128	ND
CX31	>32	>32	0.25	0.5	>256	64	32	128	128	>256	>256	>1024	64	32	8	ND
CX33	>32	>32	0.25	0.5	256	64	32	128	256	>256	>256	>1024	64	32	8	ND
CX34	>32	>32	0.25	0.25	256	64	32	256	>256	128	>256	>1024	>64	32	2	ND
CX35	>32	16	0.25	0.5	256	64	32	256	>256	256	>256	>256	64	32	2	256

...continued on next page...

ID	meropenem	imipenem	polymyxin	tigecycline	cefoxitin	cefepime	ceftazidime	cefotaxime	ceftriaxone	cefperazone	piperacillin	amikacin	ciprofloxacin	levofloxacin	minocyline	fosfomycin
CX36	32	32	0.25	0.125	128	64	32	256	>256	128	>256	0.5	>64	32	2	ND
CX37	32	32	0.125	1	128	32	16	128	128	128	256	>1024	>64	64	16	ND
CX38	16	1	0.25	0.5	4	4	32	64	64	8	8	4	>64	16	4	16
CX4	>32	16	0.25	2	256	128	256	256	256	256	>256	1	64	64	8	>256
CX40	32	32	0.25	0.5	256	64	64	128	128	>256	>256	>1024	64	32	8	ND
CX41	>32	>32	0.125	4	256	128	64	128	>256	>256	>256	2	>64	>64	128	ND
CX42	>32	>32	0.125	2	256	64	32	128	256	256	>256	2	>64	>64	128	ND
CX43	32	4	0.25	0.5	256	64	32	128	128	>256	>256	>1024	64	32	8	ND
CX44	>32	>32	0.125	4	256	128	32	128	256	256	>256	2	>64	>64	64	ND
CX45	>32	8	0.5	1	128	64	32	256	>256	256	>256	>256	64	32	2	>256
CX46	>32	>32	0.5	4	128	64	64	>256	256	>256	>256	1	>64	>64	64	>256
CX47	>32	16	0.5	1	>256	64	64	128	256	>256	>256	>256	64	32	8	>256
CX48	8	8	0.5	2	64	256	>256	>256	>256	256	>256	4	>64	64	32	>256
CX49	>32	>32	0.5	4	256	64	32	128	256	256	>256	2	>64	>64	64	>256
CX5	>32	32	0.25	4	>256	64	64	128	256	>256	>256	1	>64	32	128	>256
CX50	>32	>32	0.5	1	>256	256	256	256	>256	>256	>256	>256	64	32	8	>256
CX51	32	32	0.125	0.125	256	64	32	256	>256	256	>256	>1024	>64	32	2	ND
CX55	32	32	0.125	0.25	128	64	64	256	>256	256	>256	>1024	>64	32	2	ND
CX56	32	32	0.125	0.5	128	128	64	>256	>256	>256	>256	>1024	>64	32	8	ND
CX57	>32	16	0.5	2	128	64	32	>256	>256	>256	>256	>256	64	32	4	>256
CX58	16	16	0.25	2	>256	64	>256	256	>256	>256	>256	0.5	4	8	32	256
CX59	>32	32	0.5	2	128	128	64	>256	>256	>256	>256	>256	64	32	4	>256
CX6	>32	>32	0.25	0.5	>256	256	>256	>256	>256	>256	>256	>256	32	32	2	>256
CX60	>32	32	0.5	0.5	256	128	64	>256	>256	>256	>256	>256	64	32	2	>256
CX61	8	4	>32	4	>256	64	>256	>256	>256	>256	>256	128	1	1	2	256
CX63	>32	>32	0.5	1	256	128	64	256	>256	>256	>256	>256	>64	64	32	>256
CX65	>32	>32	0.25	1	128	64	32	>256	>256	256	>256	>1024	>64	32	4	ND
CX66	2	1	0.5	1	128	>256	32	>256	>256	>256	>256	2	4	1	4	>256
CX67	2	1	0.5	16	128	256	128	>256	>256	256	>256	2	>64	32	128	256
CX68	>32	>32	0.25	1	256	>256	256	>256	>256	>256	>256	8	32	16	4	256
CX69	>32	32	0.25	2	128	128	64	>256	>256	>256	>256	>256	64	32	4	>256
CX7	>32	>32	0.125	8	>256	128	128	256	>256	>256	>256	>256	>64	>64	64	>256
CX70	2	1	0.5	4	128	128	>256	>256	>256	64	256	2	>64	>64	32	>256
CX71	>32	32	0.5	2	256	64	64	256	>256	>256	>256	>256	64	32	4	>256
CX72	4	1	0.5	2	128	>256	256	>256	>256	64	>256	1	>64	>64	32	128
CX73	8	16	0.5	2	>256	32	>256	256	>256	>256	>256	1	1	1	8	64
CX75	32	32	0.25	0.125	256	64	32	>256	>256	256	>256	>1024	>64	32	2	ND
CX76	>32	16	0.25	1	256	64	256	128	256	256	>256	>256	32	32	4	>256
CX77	>32	16	0.5	0.5	256	64	32	256	>256	256	>256	>256	64	32	1	>256
CX78	>32	16	0.5	0.5	128	64	32	>256	>256	>256	>256	>256	>64	321	1	>256
CX79	>32	8	0.5	2	256	128	32	>256	>256	>256	>256	>256	>64	32	2	>256
CX8	>32	32	0.25	4	256	64	32	256	256	256	>256	1	>64	64	64	>256
CX80	>32	16	0.5	0.25	128	64	64	>256	>256	>256	>256	>256	64	32	1	>256
CX81	>32	16	0.5	0.25	128	64	32	256	>256	>256	>256	>256	64	32	1	>256

. . . continued on next page. . .

ID	meropenem	imipenem	polymyxin	tigecycline	cefoxitin	cefepime	ceftazidime	cefotaxime	ceftriaxone	cefperazone	piperacillin	amikacin	ciprofloxacin	levofloxacin	minocyline	fosfomycin
CX82	>32	>32	0.5	4	256	64	32	128	256	256	>256	1	>64	>64	64	>256
CX83	>32	>32	0.5	4	>256	256	128	256	>256	>256	>256	2	>64	>64	64	>256
CX84	>32	>32	0.5	0.5	256	64	32	128	256	256	>256	2	32	16	1	>256
CX85	>32	32	0.5	4	256	64	64	64	128	>256	>256	>256	>64	>64	32	>256
CX9	>32	>32	0.25	0.5	>256	64	32	128	128	>256	>256	>1024	64	32	8	ND
CX90	>32	32	0.25	1	256	128	256	256	>256	>256	>256	>256	>64	64	16	>256
CX91	>32	16	0.25	1	256	128	256	256	>256	>256	>256	>256	>64	32	8	>256
CX92	16	8	0.125	0.5	256	64	32	64	128	>256	>256	>1024	64	32	8	ND
CX94	4	4	0.25	8	>256	64	32	256	128	64	64	16	>64	>64	128	>256
CX95	1	8	0.5	8	>256	32	16	256	128	64	256	32	>64	>64	128	>256
CX96	0.125	0.125	0.5	8	64	64	256	256	>256	64	>256	4	>64	>64	128	>256
CX97	1	4	0.5	8	>256	32	16	256	128	64	256	32	>64	>64	128	>256
CX98	>32	8	0.25	2	256	64	>256	128	256	>256	>256	>256	32	32	4	>256
CX99	>32	>32	0.25	4	256	64	32	128	256	256	>256	2	>64	>64	64	128

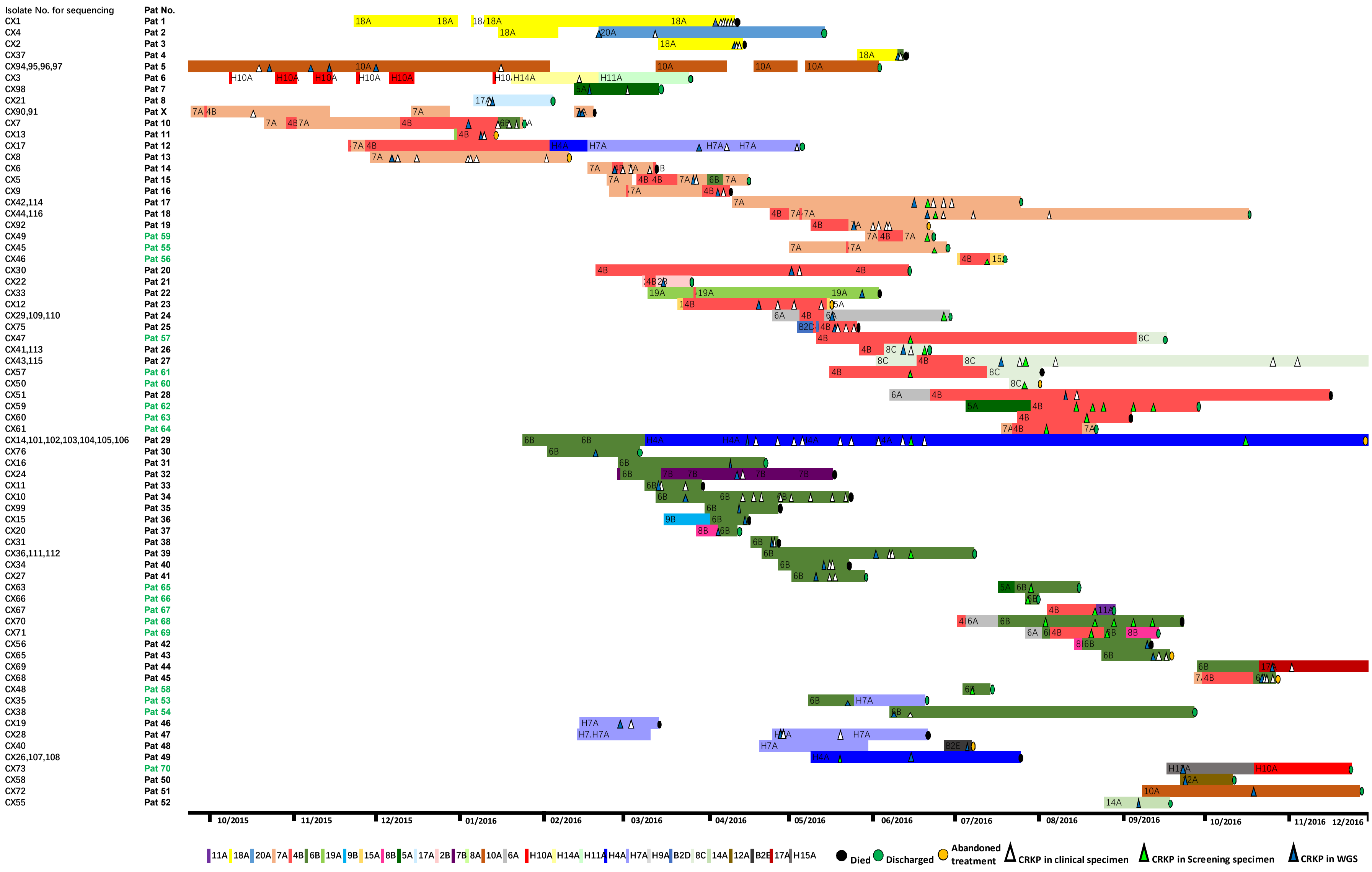

Figure S2: Patient admission information, including movement through hospital wards and time points of sampling for CRKP and for WGS. Full details of the final collection are provided in Table S1.

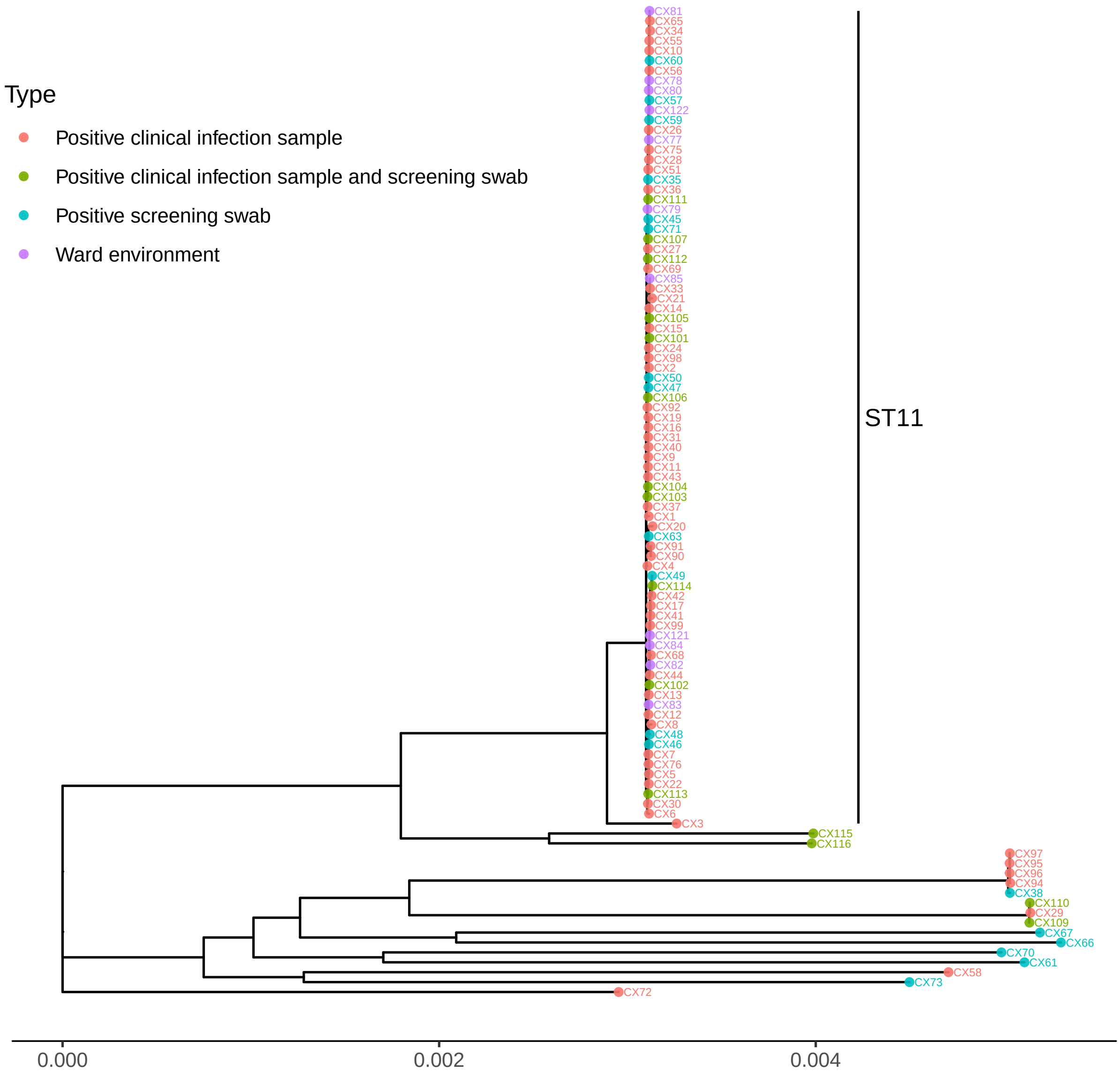

Figure S3: Core genome maximum likelihood phylogeny of 100 isolates sampled as part of the CRKP surveillance initiative. The maximum-likelihood tree is based on 4,155 shared core genes and comprises 11 different ST types. 82 closely related ST11 isolates were selected for further analyses, see Table S1. The tip colour and legend provide the type of sample from which CRKP was isolated, see Methods.

S2 Inferring a timed phylogeny and transmission networks

S2.1 BEAST analyses

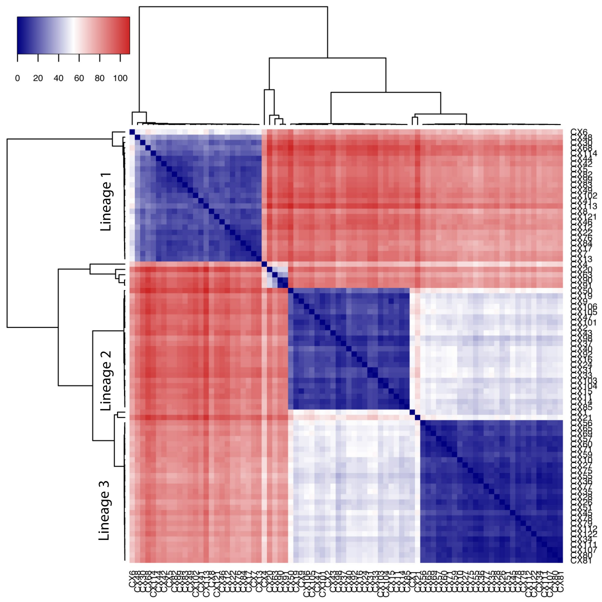

(a) Pairwise SNP difference

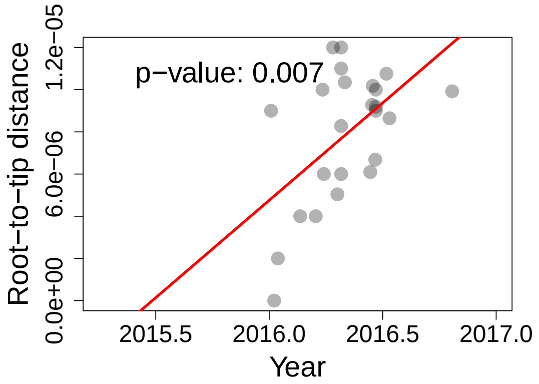

(b) Lineage 1

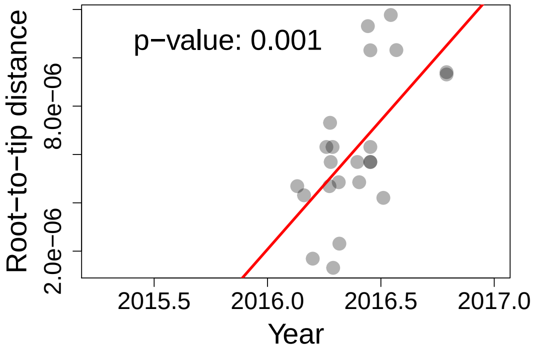

(c) Lineage 2

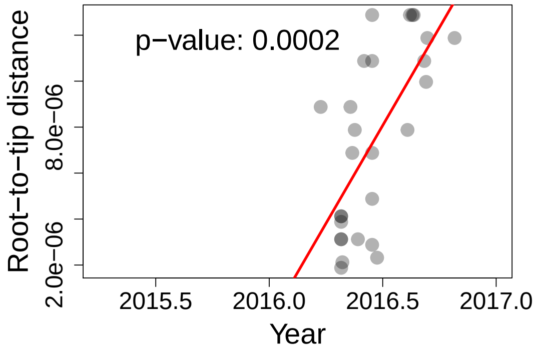

(d) Lineage 3

Figure S4: a) Pairwise SNP differences (number), as given by color scale in top left, between 82 ST11 outbreak isolates based on the recombination free chromosomal alignment b)-d) The correlation between the root-to-tip phylogenetic distance (y-axis) and time of sampling (x-axis) within Lineages 1-3, inferred using TempEst (Rambaut et al., 2016). All Lineages showed a significant correlation indicating measurable evolution during the sampling period. This justified the use of tip-dating phylogenetic approaches.

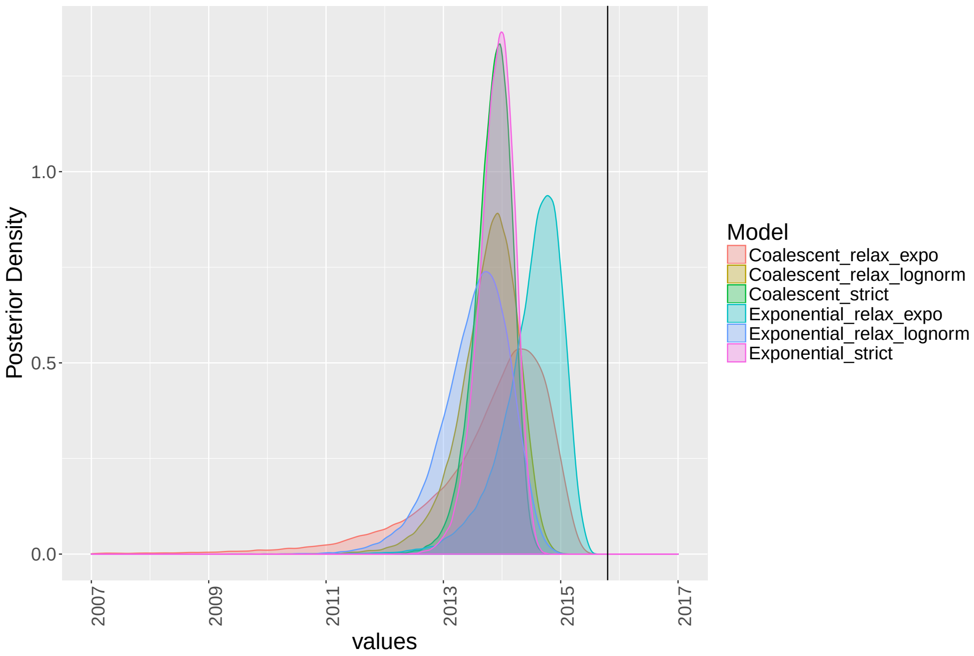

Figure S5: Inferred tMRCA under 6 alternate BEAST (?) models applied to the recombination free chromosomal alignment of 82 ST11 outbreak isolates. We applied two sets of analyses which differed in the population priors used: i) constant population size coalescent model with a strict clock (Coalescent_strict), relaxed clock with a lognormal prior (Coalescent_relax_lognorm), relaxed clock with an exponential prior (Coalescent_relax_expo) and ii) an exponential growth population model with a strict clock (Exponential_strict), relaxed clock with a lognormal prior (Exponential_relax_lognorm) and relaxed clock with an exponential prior (Exponentital_relax_expo). Median values and 5-95% HPD intervals are provided in Supplementary Table S3. The black vertical line gives the date of admission of the index patient (Patient X) which post-dates the mean tMRCA in all cases.

Table S3: Mean tMRCA, clock rates and marginal likelihoods inferred by Bayesian dating analyses applied to the recombinant free chromosomal alignment across 82 outbreak isolates. Results are provided for both the Coalescent Bayesian Skyline and Exponential growth population models tested under strict and relaxed clocks with a TN93 substitution model. The 95% Higher Posterior Densities are provided in parentheses. The marginal likelihood (ML) estimate under each model is provided based on path-sampling implemented in Beauti Path Sampler.

Population Model						
	Coalescent Bayesian Skyline			Exponential Population Growth		
Clock	Strict	Relaxed Lognormal	Relaxed Exponential	Strict	Relaxed Lognormal	Relaxed Exponential
tMRCA	2013.9(2013.1-2014.4)	2013.8(2012.6-2014.6)	2014.0(2010.6-2015.1)	2013.9(2013.2-2014.4)	2013.6(2012.2-2014.5)	2014.6(2013.2-2015.3)
Rate	3.4×10^{-6} (2.6×10^{-6} - 4.1×10^{-6})	/	/	3.4×10^{-6} (2.7×10^{-6} - 4.2×10^{-6})	/	/
ML	-7546540.903	-7546823.56	-7546518.61	-7546512.57	-7546572.86	-7546513.66

S2.2 TransPhylo analyses

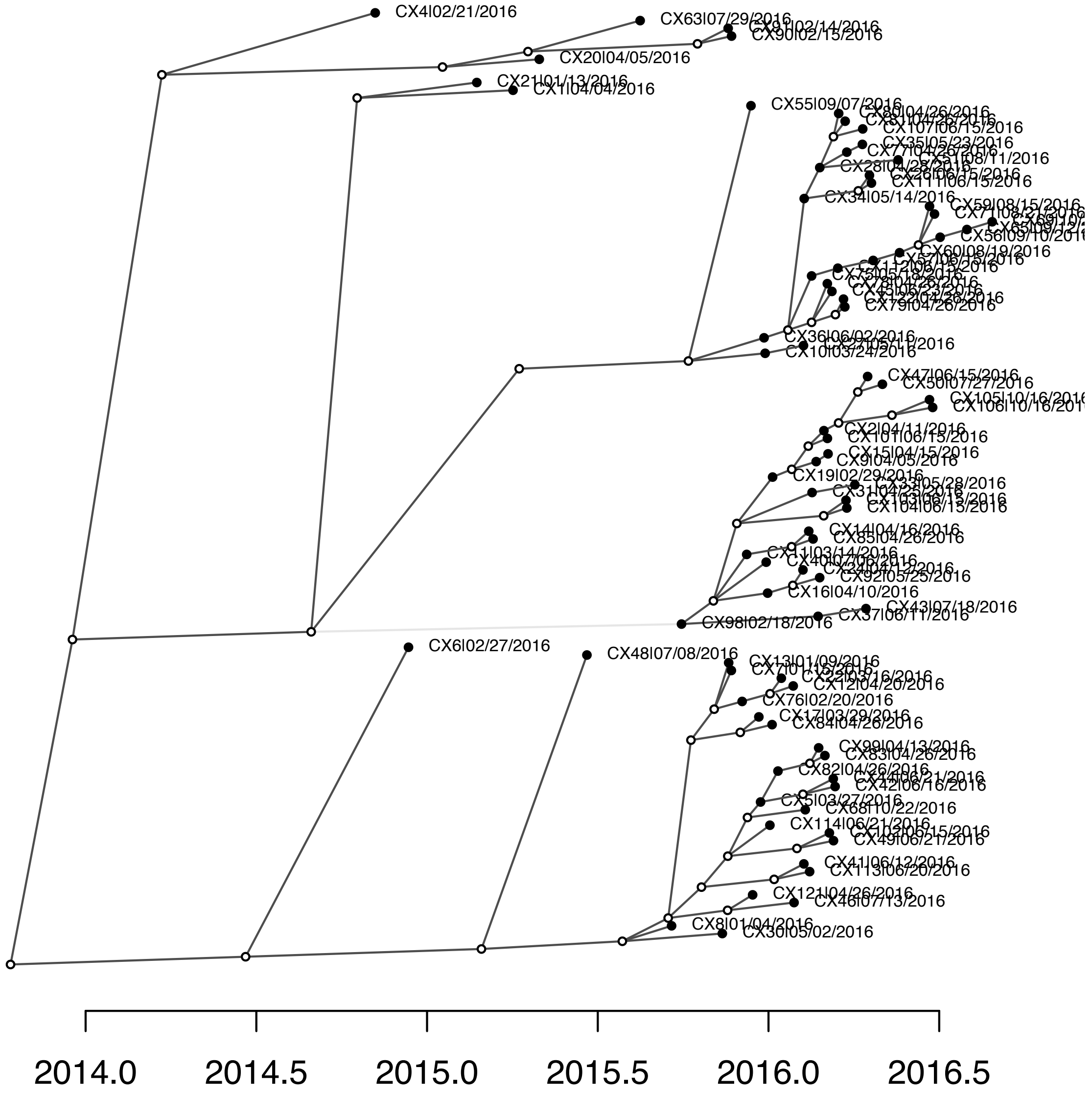

Figure S6: TransPhylo(Didelot et al., 2017) consensus transmission tree inferred across 82 ST11 isolates. The x-axis provides the mean time of infection. Filled dots represent sampled isolates and unfilled dots represent un-sampled isolates inferred to have been involved in the transmission event(s).

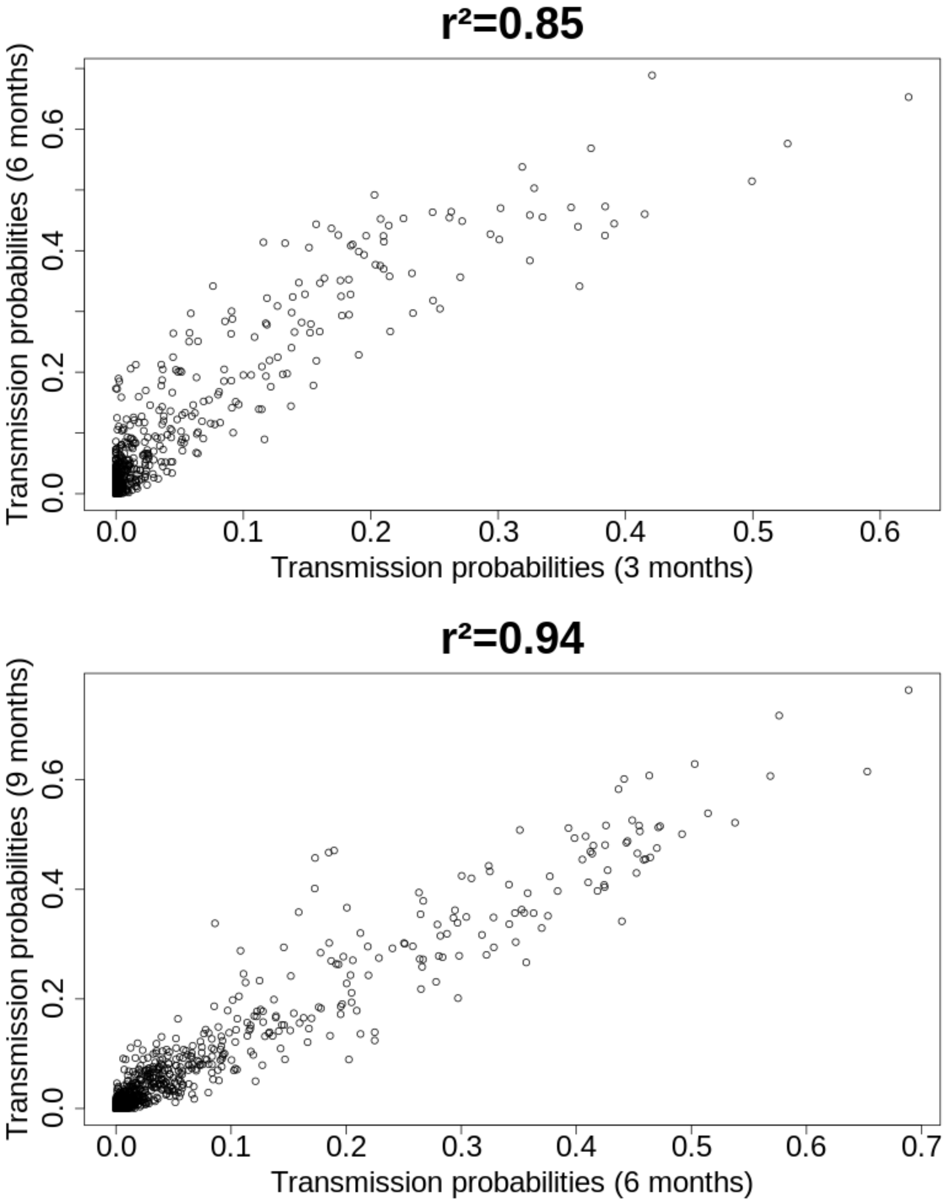

Figure S7: Pairwise correlations between the inferred probabilities of direct transmission from x to y under three independent TransPhylo runs. Each run differed in the choice of scale parameter used in the analysis, as given on the axis (scale=0.25, 3 months; scale=0.5, 6 months; scale=0.75, 9 months). The scale parameter sets the model distribution of the time taken between a transmission from a primary to secondary case. The strong correlations between runs indicates that our inferred transmission probabilities are not sensitive to different specifications.

Table S4: TransPhylo inferred probabilities of direct transmission for pairings of isolates with a probability ≥0.5. Transphylo was run on the 82 ST11 isolates. An asterisk (*) next to the transmission probability denotes those inferred transmissions where both patients directly overlapped on a ward during their admission. Two asterisks (**) next to the transmission probability denotes those patients who were admitted to the same ward within a 14 day period of each other. Isolates in the transmission pair shown in bold are from patient-associated environmental samples (see Table S1).

Transmission Pair		Patient of Isolation		Ward of Isolation		Campus of Isolation		Probability
Pair1	Pair2	Pat1	Pat2	Ward1	Ward2	Campus1	Campus2	
CX103—06/15/2016	CX104—06/15/2016	29	29	H4A	H4A	B	B	0.93
CX105—10/16/2016	CX106—10/16/2016	29	29	H4A	H4A	B	B	0.78
CX12—04/20/2016	CX22—03/16/2016	23	21	ICU(4B)	2B	A	A	0.81**
CX16—04/10/2016	CX24—04/12/2016	31	32	ICU(6B)	7B	A	A	0.50*
CX111—06/15/2016	CX26—06/15/2016	39	49	ICU(6B)	H4A	A	B	0.85*
CX10—03/24/2016	CX27—05/11/2016	34	41	ICU(6B)	ICU(6B)	A	A	0.86*
CX101—06/15/2016	CX2—04/11/2016	29	3	H4A	18A	B	A	0.64
CX31—04/25/2016	CX33—05/28/2016	38	22	ICU(6B)	19A	A	A	0.95
CX113—06/20/2016	CX41—06/12/2016	26	26	8C	8C	A	A	0.84
CX37—06/11/2016	CX43—07/18/2016	4	27	ICU(6B)	8C	A	A	0.84
CX42—06/16/2016	CX44—06/21/2016	17	18	7A	7A	A	A	0.78*
**CX121—04/26/2016**	CX46—07/13/2016	12	56	H7A	ICU(4B)	B	A	0.77
CX102—06/15/2016	CX49—06/21/2016	29	59	H4A	7A	B	A	0.85
CX47—06/15/2016	CX50—07/27/2016	57	60	ICU(4B)	8C	A	A	0.79
CX28—04/28/2016	CX51—08/11/2016	47	28	H7A	ICU(4B)	B	A	0.87
CX112—06/15/2016	CX57—06/15/2016	39	61	ICU(6B)	ICU(4B)	A	A	0.58
CX57—06/15/2016	CX60—08/19/2016	61	63	ICU(4B)	ICU(4B)	A	A	0.70**
CX56—09/10/2016	CX65—09/12/2016	42	43	ICU(6B)	ICU(6B)	A	A	0.56*
CX65—09/12/2016	CX69—10/26/2016	43	44	ICU(6B)	17A	A	A	0.89**
CX59—08/15/2016	CX71—08/21/2016	62	69	ICU(4B)	ICU(4B)	A	A	0.64*
CX112—06/15/2016	CX75—05/18/2016	39	25	ICU(6B)	ICU(4B)	A	A	0.62
CX35—05/23/2016	**CX77—04/26/2016**	53	34	ICU(6B)	ICU(6B)	A	A	0.93*
CX45—06/23/2016	**CX78—04/26/2016**	55	34	7A	ICU(6B)	A	A	0.56*
**CX122—04/26/2016**	**CX79—04/26/2016**	34	34	ICU(6B)	ICU(6B)	A	A	0.90
CX13—01/09/2016	CX7—01/15/2016	11	10	ICU(4B)	ICU(4B)	A	A	0.83**
CX107—06/15/2016	**CX81—04/26/2016**	49	34	H4A	ICU(6B)	B	A	0.76*
**CX80—04/26/2016**	**CX81—04/26/2016**	34	34	ICU(6B)	ICU(6B)	A	A	0.61
CX17—03/29/2016	**CX84—04/26/2016**	12	12	H7A	H7A	B	B	0.86
CX14—04/16/2016	**CX85—04/26/2016**	29	29	H4A	H4A	B	B	0.88
CX30—05/02/2016	CX8—01/04/2016	20	13	ICU(4B)	7A	A	A	0.78
CX90—02/15/2016	CX91—02/14/2016	X	X	7A	7A	A	A	0.93
CX24—04/12/2016	CX92—05/25/2016	32	19	7B	7A	A	A	0.76*
**CX83—04/26/2016**	CX99—04/13/2016	35	35	ICU(6B)	ICU(6B)	A	A	0.90
CX15—04/15/2016	CX9—04/05/2016	36	16	ICU(6B)	ICU(4B)	A	A	0.93*

S3 Inferring core and accessory genome structure

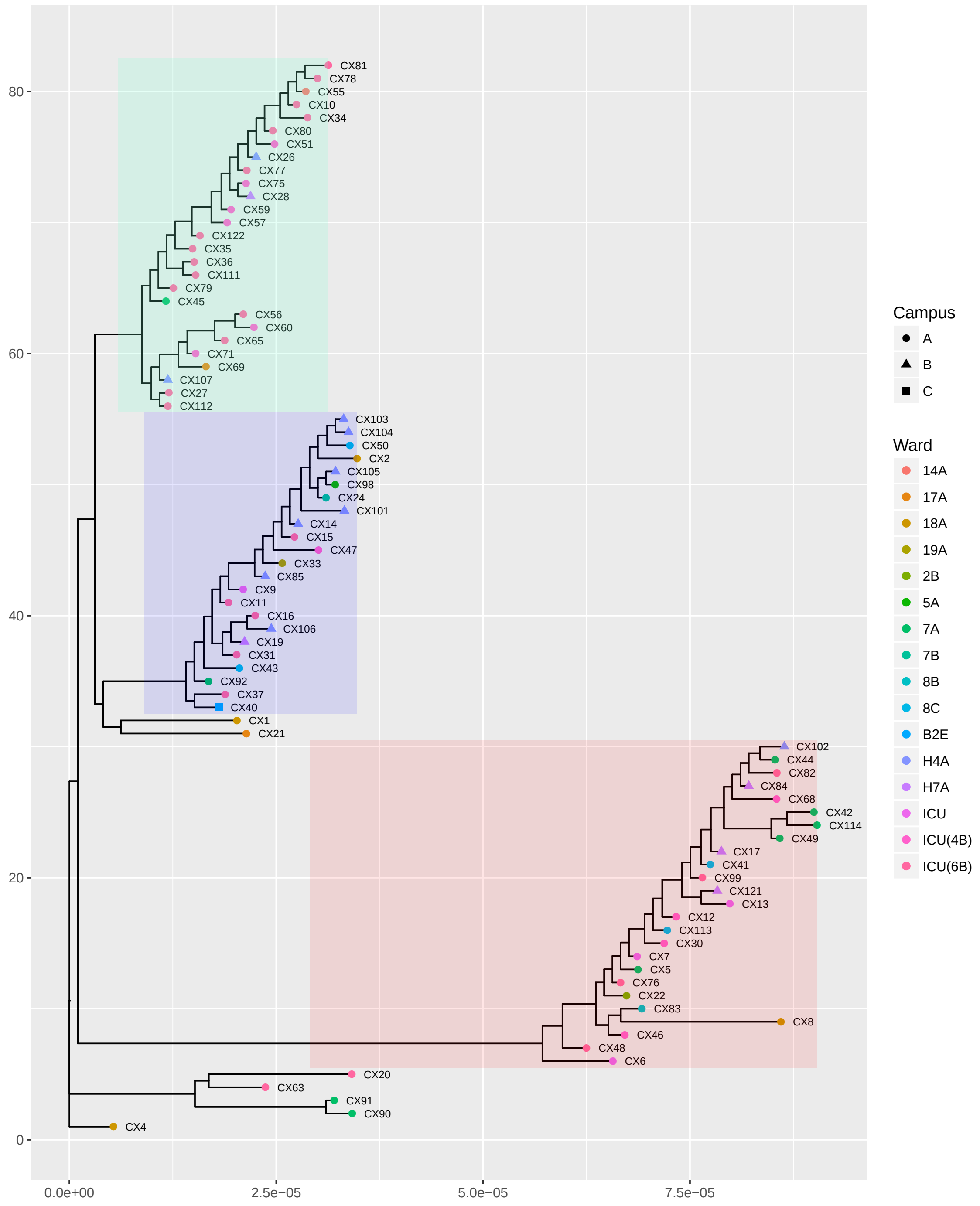

Figure S8: Core genome phylogeny of 82 ST11 outbreak isolates based on 4,652 shared core genes. Tips are colored according to the ward of isolation with symbols giving the hospital campus. The pan-genome phylogeny clusters into three main lineages as highlighted.

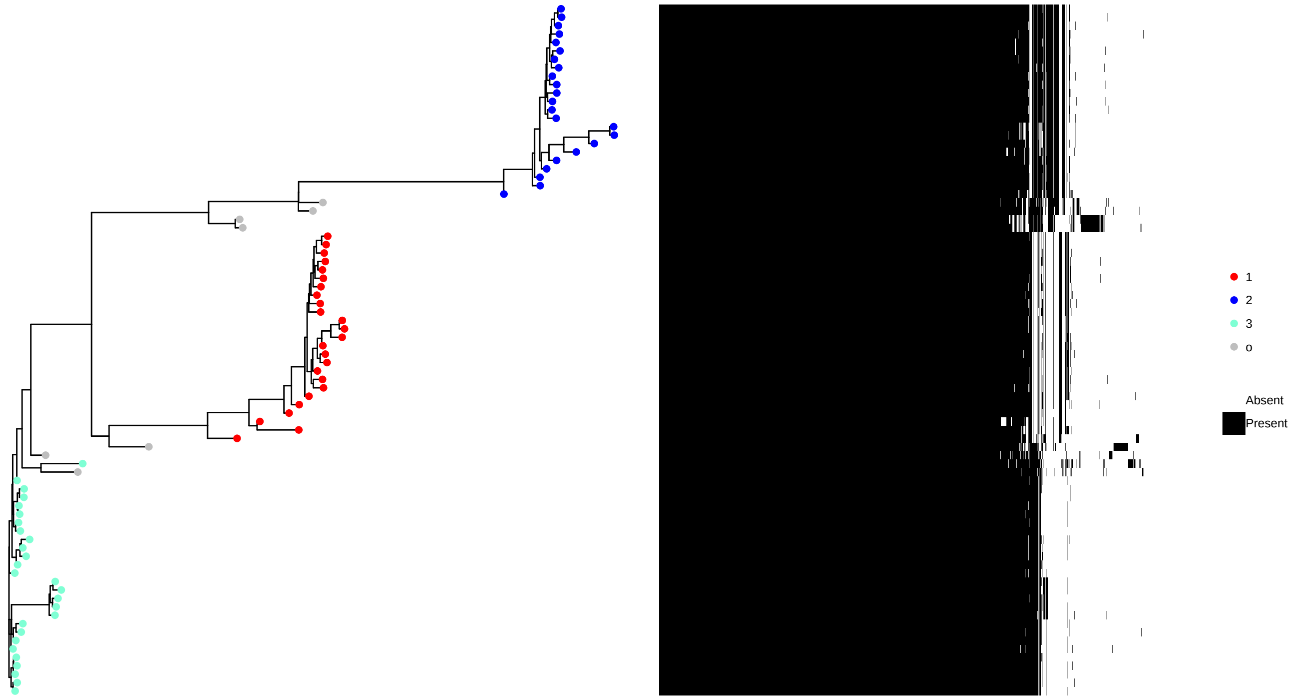

Figure S9: Left) Core and accessory distance tree based on the binary presence and absence of annotated genes. Tips are colored according to the three-lineage structure observed on the core and chromosomal alignments as given in the legend at right. Right) Presence (black) and absence (white) of annotated genes comprising the core (present in all samples) and accessory genome (non-shared) components.

Table S5: Presence of plasmid replicons as inferred by PlasmidFinder (Carattoli et al., 2014) applied to short-read *de novo* assemblies of 82 ST11 outbreak *Kpn* isolates. The frequency of each plasmid type is provided across all isolates (All) as well as within distinct phylogenetic lineages (Lineage 1-3) and among outliers (Outliers). The mean number of assignments per strain lineage is also given.

Plasmid Type	All (n=82)	Lineage 1 (n=25)	Lineage 2 (n=23)	Lineage 3 (n=27)	Outliers(n=7)
Col156	1			1	
Col(MG828)	1		1		
IncFIB(pKPHS1)	1				1
IncI2	1		1		
IncN	1				1
IncFIB(Mar)	2				2
IncX1	2		2		
ColpVC	5	2	1	2	
IncFIB(AP001918)	23	21		1	1
IncFIA	25	24		1	
IncFII	26		23	1	2
IncFIB(K)	27		23		4
IncR	28	25			3
ColRNAI	82	24	23	27	7
IncFII(pHN7A8)	82	25	23	27	7
TOTAL	307	121	97	60	28
Mean/isolate	3.74	4.84	4.22	2.22	4

S4 AMR gene copy number variation (CNV) and phenotypic resistance

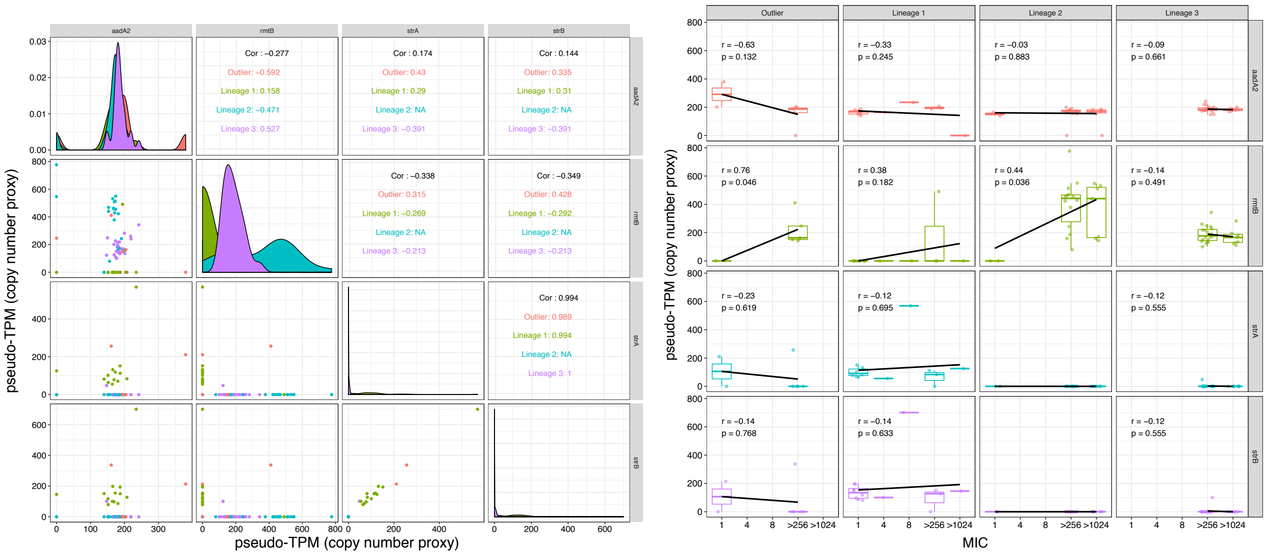

(a) Gene-gene correlations   (b) Amikacin-gene correlations

Figure S10: a) Between-gene correlations for pseudo-TPMs of four genes associated with resistance to aminoglycosides, stratified by lineage (Lineage 1:green, Lineage 2:blue, Lineage 3:purple, outliers:orange). *strA* and *strB* were highly correlated (across lineages: Spearman's $\rho = 0.998, p < 0.001$; within Lineage 1, Spearman's $\rho = 0.903, p < 0.001$), in line with their co-occurrence which has previously been observed across integrons, transposons, and broad-host-range plasmids across the Enterobacteriaceae (Sundin, 2002; Dolejska et al., 2013; Compain et al., 2014). The *strA*-*strB* genes were anti-correlated with the 16S rRNA methyltransferase *rmtB* (across lineages for *strA*: Spearman's $\rho = -0.602, p < 0.001$), which provides a higher-level of aminoglycoside resistance (Doi et al., 2004). b) Correlations between resistance gene pseudo-TPMs and measured MICs against amikacin, stratified by lineage (Lineage 1:green, Lineage 2:blue, Lineage 3:purple, outliers:orange). Trend lines and Pearson correlations are shown. Lineage 1 isolates lacked *rmtB*, and these isolates correspondingly were less resistant to amikacin. Using the EUCAST (https://mic.eucast.org) epidemiological cutoff (MIC>8.0 μg/ml), 4/14 isolates in Lineage 1 were resistant, compared to 21/23 (Lineage 2) and 27/27 (Lineage 3). Furthermore, the two isolates within Lineage 2 that were not resistant lacked *rmtB*. However, there was no difference in the pseudo-TPMs for *rmtB* comparing isolates with MIC>256μg/ml to those with MIC>1024μg/ml (Wilcoxon signed-rank test, $p = 0.42$), suggesting that the presence of *rmtB* was important but not its relative copy number i.e. that other factors dictate the different MIC values. Many isolates in Lineage 2 had approximately double the pseudo-TPM for *rmtB* of those in Lineage 3, but an additional copy was not observed in the assemblies. This would be consistent with an additional copy of the gene due to a duplicated plasmid, although this did not result in greater resistance.

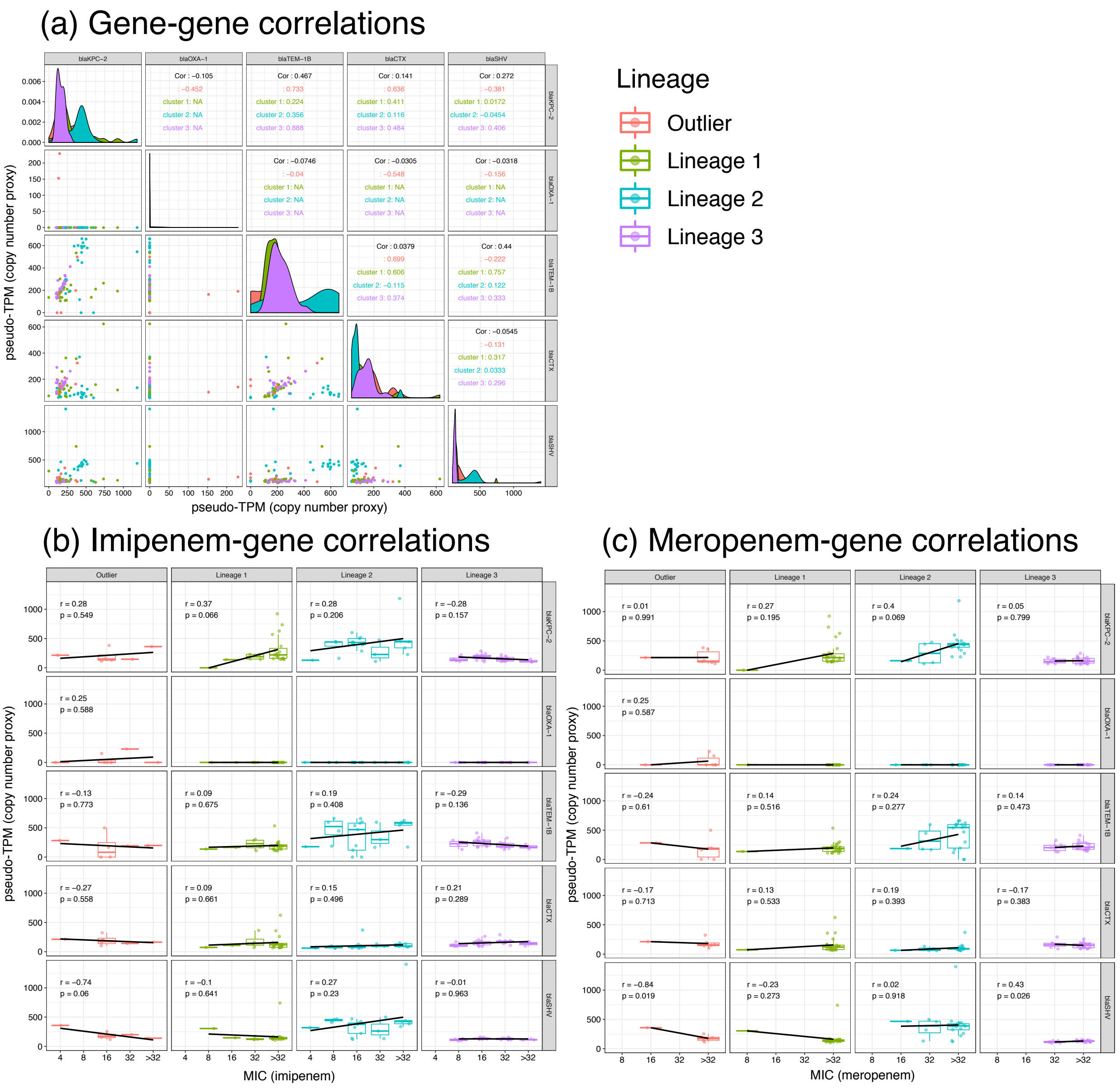

Figure S11: a) Between-gene correlations for pseudo-TPMs of two genes associated with resistance to beta-lactams, stratified by lineage (colors). Resistance to beta-lactam antibiotics (including imipenem and meropenem) can be conferred by a range of beta-lactamases. These contribute to resistance in a cumulative manner, contrasting with the anti-correlation as observed for aminoglycoside resistance genes with different mechanisms (Supplementary Figure S10). b)-c) Correlations between resistance gene pseudo-TPMs and measured MICs of (b) imipenem and (c) meropenem, stratified by lineage (colors). Trend lines and Pearson correlations are shown. blaKPC-2 was associated with increased MICs to imipenem and meropenem in Lineage 1 and Lineage 2 but not Lineage 3. Not shown are beta-lactamase genes that do not hydrolyze carbapenems, which were present in the dataset (e.g. *blaOXA-1*, *blaSHV*, *blaTEM-1*) but for which we observed no significant correlations with imipenem or meropenem MICs, as expected.

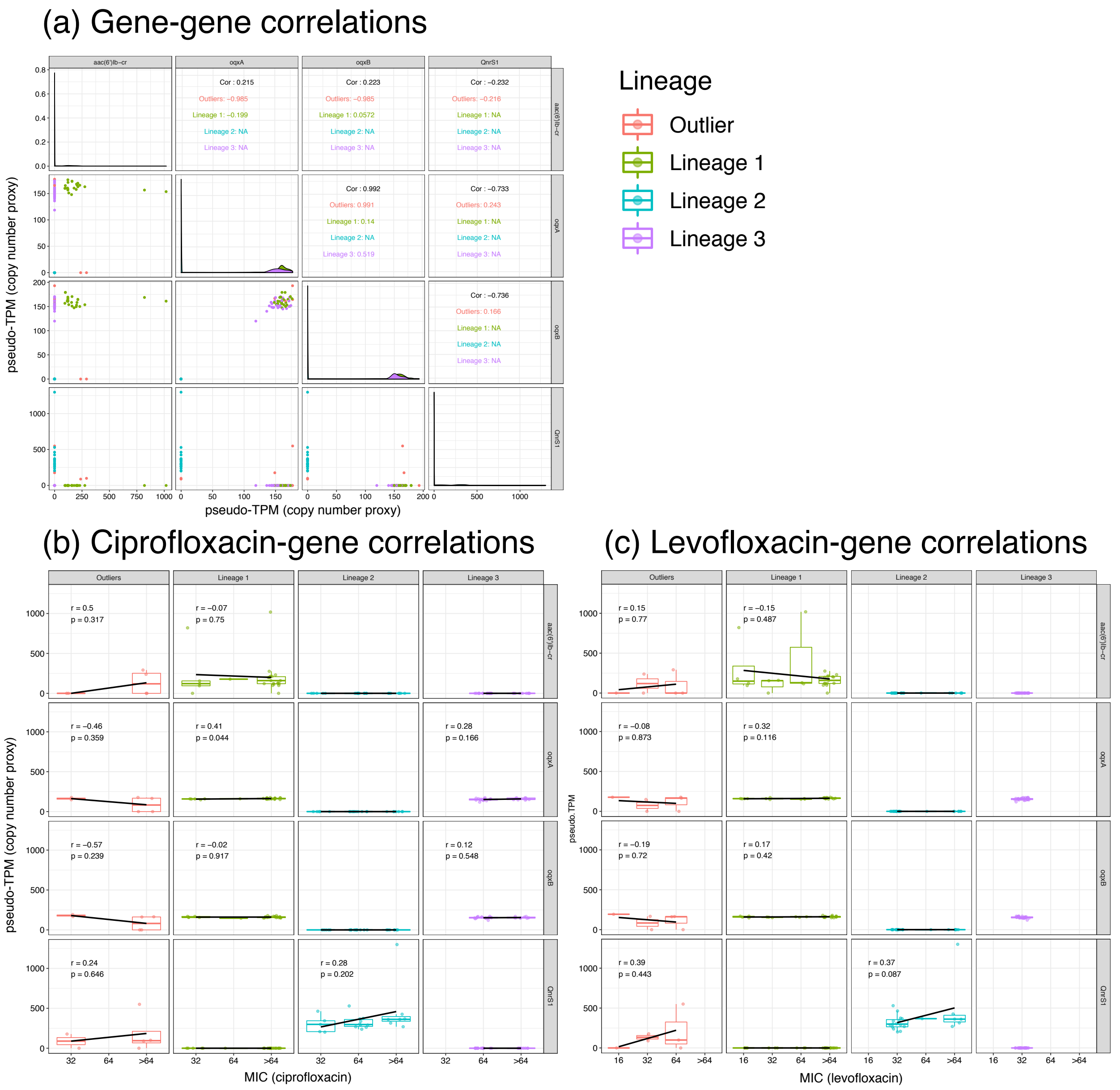

Figure S12: a) Between-gene correlations for pseudo-TPMs of four genes associated with resistance to quinolones, stratified by lineage (colors). Quinolone resistance is additionally linked to the quinolone resistance-determining regions (QRDR) in the *gyrA* and *parC* genes (Jacoby, 2005). Comparing sequences to the genes in *K. pneumoniae* ATCC 13883 reference strain, all isolates had common mutations at the 83rd and 87th resistance 'hotspots' of *gyrA* (S83I and D87G). In *parC* one of the mutation hotspots was mutated (S80I). All isolates correspondingly were resistant according to EUCAST epidemiological cutoffs (ciprofloxacin MIC>0.125 μg/ml; levofloxacin MIC>0.25 μg/ml). The presence of multiple fluoroquinolone resistance determinants has been reported to be cumulative, including both point mutations and additional genes (Ruiz et al., 2012; Al-Marzooq et al., 2014). The efflux system of two genes *oqxAB* was present in Lineages 1 and 3 but not in Lineage 2, which had *QnrS1* present instead. b)-c) Correlations between resistance gene pseudo-TPMs and measured MICs of (b) ciprofloxacin and (c) levofloxacin. Trend lines and Pearson correlations are shown. The pseudo-TPM (copy number) of *QnrS1* was associated with increased MICs for levofloxacin and ciprofloxacin in Lineage 2 and in the outlying isolates.

S5 Plasmid assemblies

Table S6: PacBio assemblies providing the isolate ID and lineage assignment (outlier O, 1, 2, 3) together with assembly sizes (in base pairs). The method applied to generate each assembly is provided. All use UniCycler (Wick et al., 2017) (abbreviation UC) but differ in whether short-read data is used to inform the assembly (hybrid), or only long-read data is used (PacBio_only) in normal (default) or bold (bold) mode. In some instances raw PacBio reads were filtered for quality using FiltLong. For CX45, additional polishing was performed following assembly with Racon (Vaser et al., 2017). Most plasmids were reported as circularised by UniCycler. * denotes those plasmids which were manually circularised, based on the presence of exactly overlapping sequence ends in the assembly. '+' denotes where the plasmid has been assembled using additional information from the short-read only *de novo* assembly. The chromosomes of CX45 and CX77 were not circularised and in both cases assembled as two linear contigs.

ID	Lineage	Method	Chromosome	Virulence	$bla_{\text{KPC-}}$	$bla_{\text{KPC+}}$	≈10Kb Plasmid
CX90	O	UC_raw_hybrid	5,412,826	284,901	123,558	116,106	10,060
CX20		UC_raw_hybrid	5,477,860	/	243,743	128,569	10,060
CX41	1	UC_Filtlong_PacBio_only	5,543,726	/	126,359	53,491	10,263
CX16	2	UC_raw_hybrid	5,447,232	/	101,140	295,742*	10,063
CX104		UC_Filtlong_PacBio_only	5,575,620	/	201,030	124,464*	10,062+
CX105		UC_PacBio_only	5,575,842	/	36,801*	348,763*	10,060+
CX45	3	UC_PacBio_only_bold_plus_racon	4346009,1238371	/	/	135,777*	11,972+
CX77		UC_raw_hybrid	3405931,2070976	/	/	135,788	11,970

Table S7: Plasmid type assignments of plasmids assembled using PacBio long-read sequencing technology. Plasmid types were assigned using PlasmidFinder (Carattoli et al., 2014) based on the presence of specific replicon sequences with >95% percentage identity to the reference, as also shown in main text Figure 3. All isolates carried a colRNAI plasmid.

Isolate	Lineage	Virulence	$bla_{\text{KPC-}}$	$bla_{\text{KPC+}}$	≈10Kb Plasmid
CX90	O	IncFIB(Mar)	IncFIB(K)	IncFII	colRNAI
CX20		/	IncFIB(K),IncFII	IncFII, IncR	colRNAI
CX41	1	/	IncFIB, IncFII	IncFIA	colRNAI
CX16	2	/	IncFII	IncFIB(K)	colRNAI
CX104		/	IncFIB(K)	IncFII	colRNAI
CX105		/	IncR	IncFIB(K),IncFII	colRNAI
CX45	3	/	/	IncFII	colRNAI
CX77		/	/	IncFII	colRNAI

S5.1 Plasmid copy number variation

Table S8: For each plasmid assembled using PacBio long read sequencing technology (Table S6), we provide the average coverage of that same isolate's short-read data mapped back to the plasmid (point estimate). This provides a proxy for plasmid copy number. We also provide the 95% CI in parentheses of the mean coverage across each of our 82 ST11 short-read samples mapped back to that same plasmid. A '/' indicates the absence of this plasmid in the sequenced isolate assembly. We observed considerable variability in coverage across the outbreak cohort, with instances of clear plasmid loss and gains, as also highlighted in main text Figures 3 and 4.

Isolate	Lineage	Virulence	*bla*$_{\text{KPC-}}$	*bla*$_{\text{KPC+}}$	≈10Kb Plasmid
CX90	O	1.32(0-0)	0.48(0-1.07)	0.89(0.44-2.64)	1.54(0.95-6.66)
CX20		/	0.68(0-0.86)	0.62(0.44-2.02)	0.94(0.94-6.19)
CX41	1	/	0.99(0.44-1.72)	0.92(0.46-2.69)	2.1(1.04-6.92)
CX16	2	/	0.48(0-1.07)	3.14(0.34-3.14)	2.09(0.95-6.49)
CX104		/	0.66(0-1.08)	3.65(0.48-4.58)	8.96(1.63-9.99)
CX105		/	1.35(0-7.72)	1.55(0.35-2.11)	11.97(1.97-11.97)
CX45	3	/	/	0.99(0.72-3.15)	2.97(1.45-6.18)
CX77		/	/	1.42(0.74-3.29)	3.1(1.49-6.34)

Table S9: For each plasmid assembled using PacBio long read sequencing technology (Table S6), we provide the percentage (%) of the plasmid covered by that same isolate's short-read data mapped back to the plasmid (point estimate). We also provide the 95% CI in parentheses of the mean percentage of the plasmid covered across each of our 82 ST11 short-read samples mapped back to that same plasmid. A '/' indicates the absence of this plasmid in the sequenced isolate assembly. As with coverage (see main text Figure 3 and 4), we observed considerable variability in the percentage of the genome covered for different plasmids.

Isolate	Lineage	Virulence	*bla*$_{\text{KPC-}}$	*bla*$_{\text{KPC+}}$	≈10Kb Plasmid
CX90	O	100 (5.5-10.5)	100 (10.9-99.8)	100 (52.1-98.9)	100 (100-100)
CX20		/	100(10.5-80.7)	100 (66.1-86.2)	100 (100-100)
CX41	1	/	89.8 (40.3-92.3)	91.7 (52.4-86.9)	96.8 (96.4-98.2)
CX16	2	/	100 (34.5-100)	100 (37.9-100)	100 (100-100)
CX104		/	94.2 (4.2-92.3)	92.2 (59.4-91.3)	100 (100-100)
CX105		/	92.3 (2.9-70.5)	87.6 (28.0-87.0)	100 (100-100)
CX45	3	/	/	89.8 (70.3-100)	100 (100-100)
CX77		/	/	100 (85.6-100)	100 (100-100)

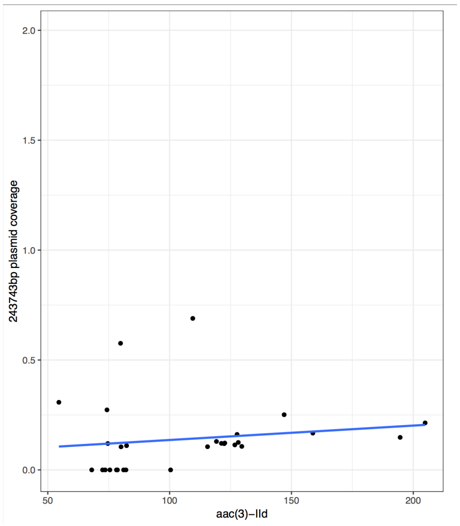

(a) *aac(3)-lld*

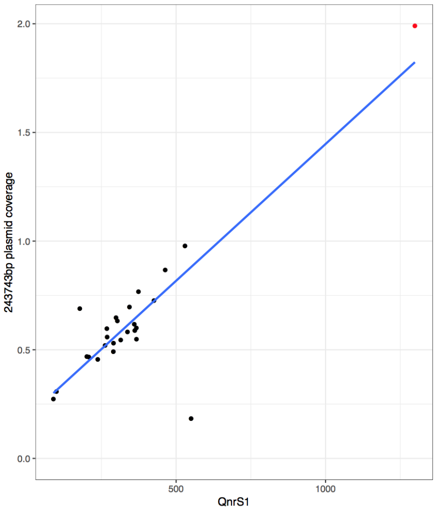

(b) *qnrS*

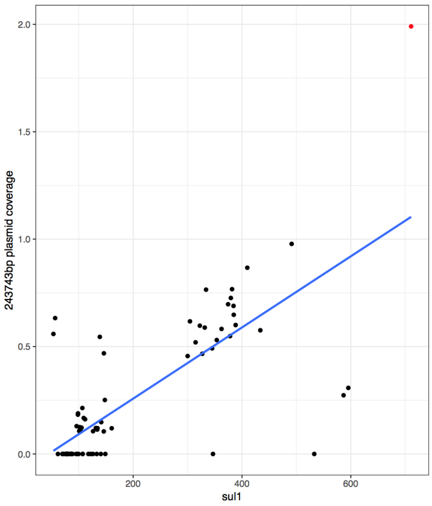

(c) *sul1*

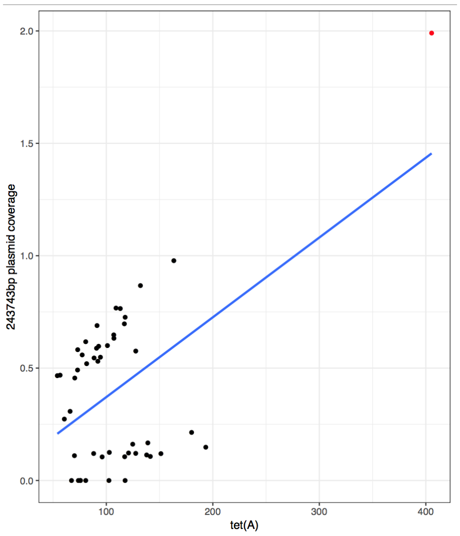

(d) *tetA*

Figure S13: Relationship between pseudo TPMs (see Methods) estimated for *aac(3)-lld*, *qnrS*, *sul1*, *tetA* (x-axis) to the coverage of short-read sequence data mapped against the 243,743bp complete plasmid assembled from CX20 (y-axis).

S5.2 Accessory genome mobility

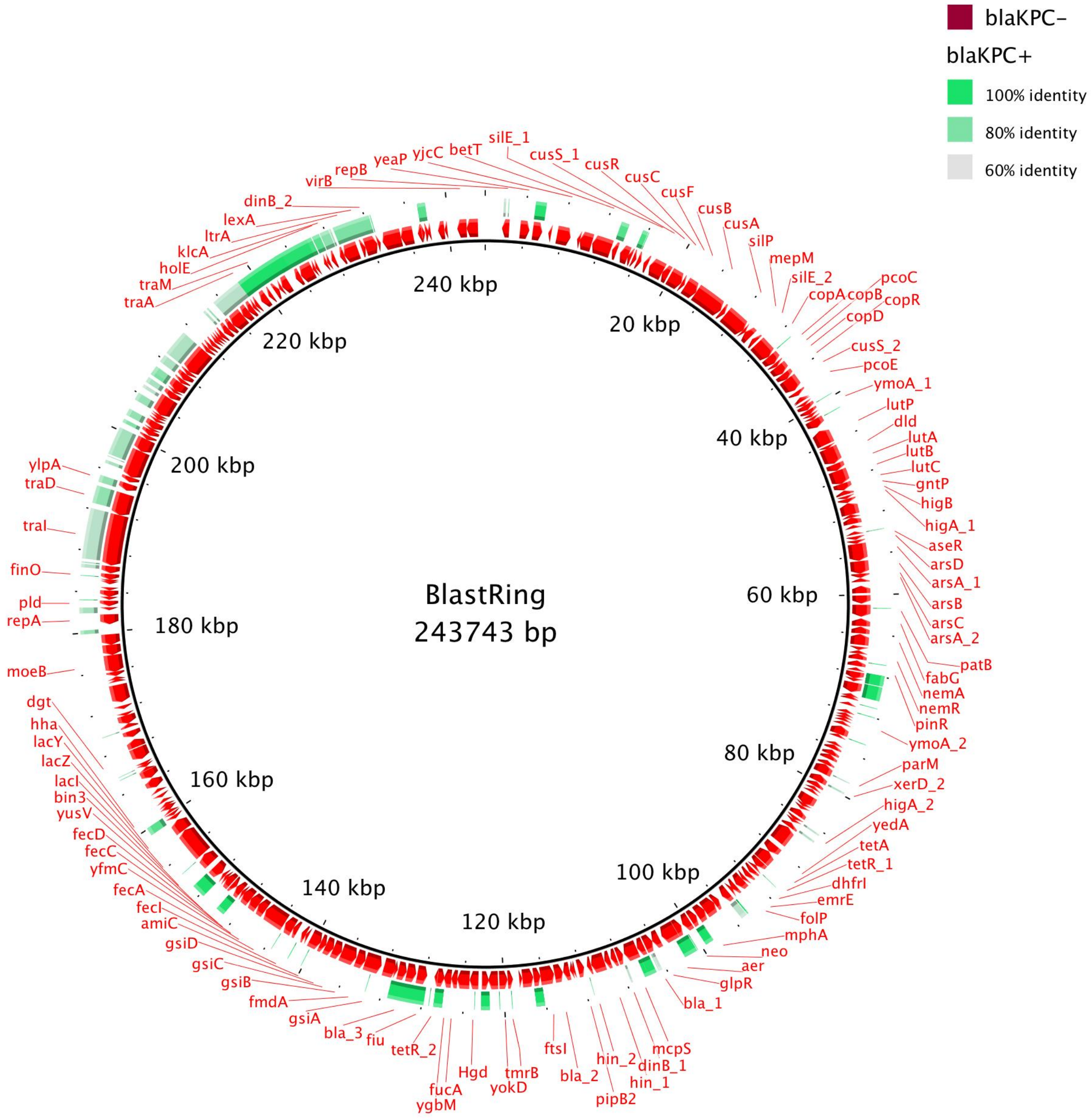

Figure S14: Blast ring (Alikhan et al., 2011) of % identity between two plasmids assembled using long-read data from CX20. The $bla_{\text{KPC+}}$ plasmid (outer ring; green), shares a portion of its genome with the other large $bla_{\text{KPC-}}$ plasmid assembled in this strain (inner ring; red). This region contains transfer genes *traI*, *traD*, *traA*, *traM* and may represent a region of putative recombination between plasmids in circulation in the same strain.

S5.3 *bla*$_{\mathrm{KPC}}$ mobility

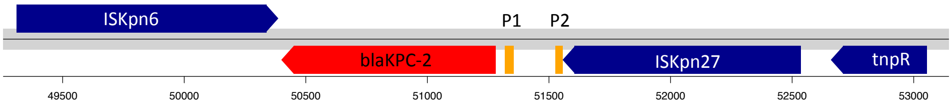

(a)

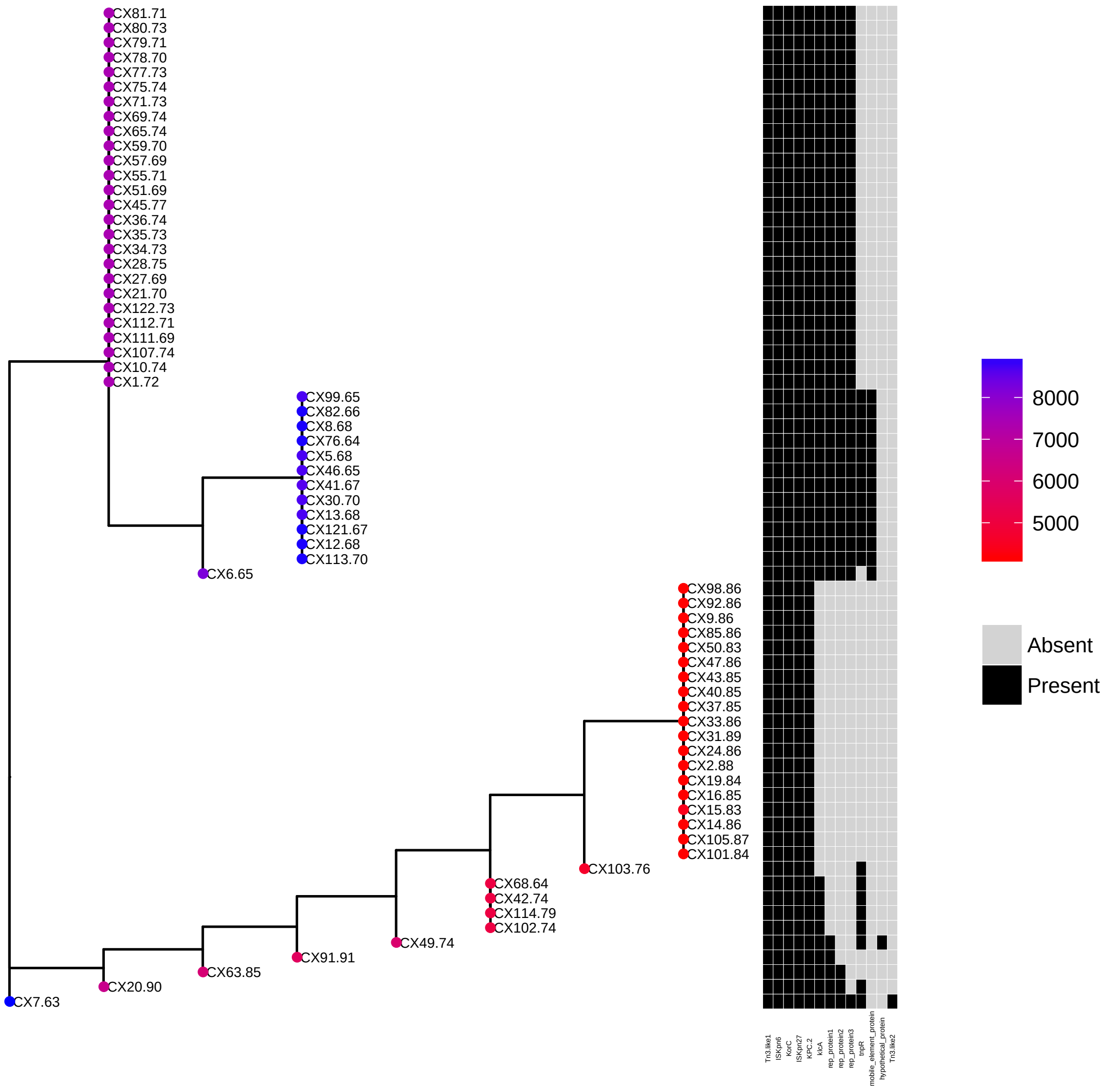

(b)

Figure S15: a) Genomic structure of the *bla*$_{\mathrm{KPC+}}$ TN*1722* type transposon which was highly conserved across our 82 ST11 isolates. b) Gene presence absence binary phylogeny of genes annotated on *bla*$_{\mathrm{KPC+}}$ contigs. The annotation was compared across 68 ST11 isolates for which there was a region at least >1200bp long either side of *bla*$_{\mathrm{KPC}}$. The color scale gives the contig lengths.

References

Al-Marzooq, F., Mohd Yusof, M.Y. and Tay, S.T. Molecular analysis of ciprofloxacin resistance mechanisms in Malaysian ESBL-producing Klebsiella pneumoniae isolates and development of mismatch amplification mutation assays (MAMA) for rapid detection of gyrA and parC mutations. *BioMed research international*, 2014:601630, 2014. ISSN 2314-6141. doi: 10.1155/2014/601630.

Alikhan, N.F., Petty, N.K., Ben Zakour, N.L. and Beatson, S.A. BLAST Ring Image Generator (BRIG): simple prokaryote genome comparisons. *BMC Genomics*, 12(1):402, dec 2011. ISSN 1471-2164. doi: 10.1186/1471-2164-12-402.

Carattoli, A., Zankari, E., García-Fernández, A., Voldby Larsen, M., Lund, O., Villa, L., Møller Aarestrup, F. and Hasman, H. <i>In Silico</i> Detection and Typing of Plasmids using PlasmidFinder and Plasmid Multilocus Sequence Typing. *Antimicrobial Agents and Chemotherapy*, 58 (7):3895–3903, jul 2014. ISSN 0066-4804. doi: 10.1128/AAC.02412-14.

Compain, F., Frangeul, L., Drieux, L., Verdet, C., Brisse, S., Arlet, G. and Decré, D. Complete nucleotide sequence of two multidrug-resistant IncR plasmids from Klebsiella pneumoniae. *Antimicrobial agents and chemotherapy*, 58(7):4207–10, jul 2014. ISSN 1098-6596. doi: 10.1128/AAC.02773-13.

Didelot, X., Fraser, C., Gardy, J. and Colijn, C. Genomic infectious disease epidemiology in partially sampled and ongoing outbreaks. *Molecular Biology and Evolution*, 34(4):msw075, jan 2017. ISSN 0737-4038. doi: 10.1093/molbev/msw275.

Doi, Y., Yokoyama, K., Yamane, K., Wachino, J.I., Shibata, N., Yagi, T., Shibayama, K., Kato, H. and Arakawa, Y. Plasmid-mediated 16S rRNA methylase in Serratia marcescens conferring high-level resistance to aminoglycosides. *Antimicrobial agents and chemotherapy*, 48(2):491–6, feb 2004. ISSN 0066-4804.

Dolejska, M., Villa, L., Dobiasova, H., Fortini, D., Feudi, C. and Carattoli, A. Plasmid Content of a Clinically Relevant Klebsiella pneumoniae Clone from the Czech Republic Producing CTX-M-15 and QnrB1. *Antimicrobial Agents and Chemotherapy*, 57(2):1073–1076, feb 2013. ISSN 0066-4804. doi: 10.1128/AAC.01886-12.

Jacoby, G.A. Mechanisms of Resistance to Quinolones. *Clinical Infectious Diseases*, 41(Supplement_2): S120–S126, jul 2005. ISSN 1537-6591. doi: 10.1086/428052.

Rambaut, A., Lam, T.T., Max Carvalho, L. and Pybus, O.G. Exploring the temporal structure of heterochronous sequences using TempEst (formerly Path-O-Gen). *Virus evolution*, 2(1):vew007, jan 2016. ISSN 2057-1577. doi: 10.1093/ve/vew007.

Ruiz, J., Pons, M.J. and Gomes, C. Transferable mechanisms of quinolone resistance. *International Journal of Antimicrobial Agents*, 40(3):196–203, sep 2012. ISSN 09248579. doi: 10.1016/j.ijantimicag.2012.02.011.

Sundin, G.W. Distinct Recent Lineages of the strA - strB Streptomycin-Resistance Genes in Clinical and Environmental Bacteria. *Current Microbiology*, 45(1):63–69, jul 2002. ISSN 0343-8651. doi: 10.1007/s00284-001-0100-y.

Vaser, R., Sović, I., Nagarajan, N. and Šikić, M. Fast and accurate de novo genome assembly from long uncorrected reads. *Genome research*, 27(5):737–746, jan 2017. ISSN 1549-5469. doi: 10.1101/gr.214270.116.

Wick, R.R., Judd, L.M., Gorrie, C.L. and Holt, K.E. Unicycler: Resolving bacterial genome assemblies from short and long sequencing reads. *PLOS Computational Biology*, 13(6):e1005595, jun 2017. ISSN 1553-7358. doi: 10.1371/journal.pcbi.1005595.
